# The impact of an oral purified microbiome therapeutic on the gastrointestinal microbiome

**DOI:** 10.1038/s41591-025-04076-w

**Published:** 2026-01-05

**Authors:** Jessica A. Bryant, Marin Vulić, Emily A. Walsh, Edward G. Allen, Nicholas J. Beauchemin, Meghan E. Chafee, Liyang Diao, Kathrin Fenn, Kara A. Ford, Brooke R. Hasson, Kevin D. Litcofsky, Mary-Jane Lombardo, Asuncion Martinez, Edward J. O’Brien, Timothy J. Straub, Sean M. Sykes, Lisa Faye Marshall, Jonathan A. Winkler, Barbara H. McGovern, Christopher B. Ford, Jennifer R. Wortman, Matthew R. Henn

**Affiliations:** Seres Therapeutics, Cambridge, MA USA

**Keywords:** Microbiome, Pharmacology

## Abstract

VOWST (VOWST oral spores, VOS; fecal microbiota spores, live-brpk, formerly SER-109) is an FDA-approved, orally administered consortium of purified Firmicutes spores developed to prevent recurrent *Clostridioides difficile* infection (CDI). Although 86.7% (26/30) of patients with recurrent CDI did not experience a subsequent recurrence over 8 weeks in an open-label phase 1b study, a subsequent double-blind phase 2 study (NCT02437487) did not demonstrate a significant benefit over placebo (rate of recurrence at 8 weeks in SER-109 versus placebo: 44.1% versus 53.3%). These discordant outcomes were hypothesized to be due to suboptimal dosing. This hypothesis was addressed in a pivotal phase 3 trial (NCT03183128) using an approximately tenfold higher dose. In phase 3, only 12% of VOS-treated patients versus 40% of placebo patients recurred by week 8 (relative risk 0.32, *P* < 0.001). Here in this follow-up post hoc analysis, across-trial comparisons confirmed that the higher, efficacious phase 3 dose is associated with improved pharmacokinetics, assessed by VOS engraftment (patients with available samples: phase 1b: 28, phase 2: 79, phase 3: 170). In-depth phase 3 analyses revealed that VOS significantly altered microbial composition, significantly enriching the diversity and abundance of Firmicutes species and reducing the prevalence and abundance of *C. difficile* and opportunistic pathogens (for example, *Enterobacteriaceae* species). Consistent with these taxonomic changes, significant changes in key bioactive metabolites were observed, including depletion of conjugated and deconjugated primary bile acids, enrichment of secondary bile acids and increases in short-chain and medium-chain fatty acids. In vitro, VOS batches produced these *C. difficile*-inhibiting metabolites. These findings on the pharmacology of VOS underscore the importance of rapidly restoring key protective functions of the microbiome in patients with recurrent CDI to achieve durable prevention of recurrence, as observed in the phase 3 study; they also highlight the need to include the microbiome in the clinical management of CDI. ClinicalTrials.gov registrations: NCT02437487 and NCT03183128.

## Abstract (web summary)

An exploratory analysis of the phase 3 ECOSPOR III trial shows that a higher dosage of the oral microbiome therapeutic VOWST led to enhanced pharmacokinetics, increased species engraftment and altered microbiome and metabolite profiles, providing mechanistic insights into how it may prevent *Clostridioides difficile* infection recurrence.

## Main

The debilitating diarrhea of CDI is driven by bacterial toxin production leading to inflammatory colitis. *C. difficile* is a unique pathogen in that 20–30% of patients have recurrent infection despite antibiotics, due to persistent microbiome disruption^1,2^. As a result, management of recurrent CDI (rCDI) with antibiotics alone has historically led to high rates of recurrence with risk of hospitalization and death. Although the role of the gastrointestinal microbial community in preventing *C. difficile* recurrence is complex, two dominant mechanisms have been postulated to suppress *C. difficile* pathogenesis. Microbe-mediated conversion of conjugated primary bile acids to secondary bile acids is inhibitory to *C. difficile* spore germination and growth^3–7^. Additionally, microbial production of short-chain or medium-chain fatty acids may inhibit *C. difficile* growth and suppress colonic inflammation and maintain epithelial barrier integrity^8–10^. However, in patients with rCDI, the gastrointestinal microbiome is characterized by antibiotic-mediated depletion of commensal bacteria, reduced bacterial richness and consequently low concentrations of secondary bile acids and short-chain fatty acids^4,11^. Although it has been proposed that fecal microbiota transplant (FMT) products are required to restore *C. difficile*-inhibitory microbiome function, spores produced by members of the Firmicutes phylum (renamed Bacillota) are sufficient to restore *C. difficile* colonization resistance^12^.

Fecal microbiota spores, live-brpk (VOWST, formerly SER-109 and hereafter referred to as VOS), is the first FDA-approved, orally administered, microbiome-based live biotherapeutic product. VOS comprises purified Firmicutes bacterial spores, manufactured by processing fecal matter from rigorously screened heathy donors with ethanol to kill organisms that are not spores, followed by filtration steps to remove solids and residual ethanol^13,14^. In contrast to FMT, VOS spore purification removes many stool components, including vegetative Proteobacteria cells. VOS manufacturing steps were previously shown to inactivate bacterial pathogens, fungi, parasites and viruses, including a model coronavirus, mitigating risk of transmission of known and emerging infectious agents^15–17^. In a phase 3, double-blind, placebo-controlled randomized trial in patients with a history of rCDI, VOS was superior to placebo in preventing recurrence at 8 weeks and was well tolerated^12^. Most CDI recurrences occurred within 4 weeks of antibiotic discontinuation, highlighting the need for rapid restoration of microbiome functions associated with the prevention of rCDI.

Critical decision-making in the development of VOS was informed by assessment of pharmacokinetics and pharmacodynamics^13,18^. Engraftment (that is, pharmacokinetics) was defined as the number of newly appearing VOS dose species in the gastrointestinal tract, and pharmacodynamics is the resulting changes in the microbiota and microbe-associated metabolites^13,19^. An open-label, dose-ranging, investigator-sponsored phase 1b study demonstrated low recurrence rates^18^ and similar VOS engraftment across dose levels, leading to a subsequent randomized, placebo-controlled phase 2b study with a lower fixed dose (NCT02437487). This phase 2b study missed its primary efficacy endpoint^13^. A follow-up pharmacological assessment led to the hypothesis that these markedly discordant results were due to differences in VOS dosing titer and frequency, resulting in reduced engraftment and lower concentrations of secondary bile acids. An approximately 10-fold higher dose was given over 3 days in the phase 3 trial (NCT03183128), which demonstrated superiority of VOS to placebo in preventing rCDI^12^. In light of the successful phase 3 study, we compared the pharmacokinetics of the phase 3 trial to the phase 1b and phase 2 trials to address the hypothesis that different dosing regimens across the VOS clinical development program led to different clinical results. We then performed an in-depth post hoc analysis of the community and functional microbiome changes in the phase 3 trial to derive insights into the mechanism of action of VOS.

## Results

### Patient disposition

**Extended Data Fig. 1:**
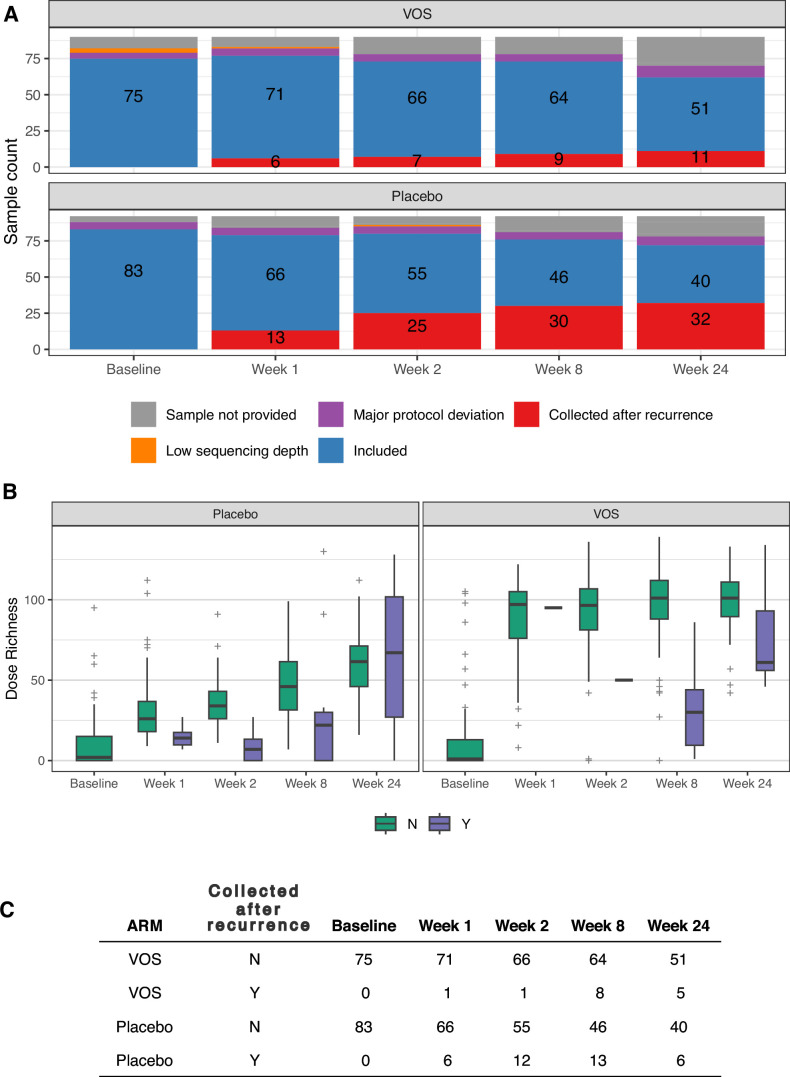
Phase 3 trial stool samples inclusion by treatment arm and timepoint. (**a**) Blue segments correspond to the number of subject samples available for analysis. Orange indicates samples where MGX sequencing produced fewer reads than the minimum threshold. Purple indicates samples excluded due to a trial protocol deviation. Red segments correspond to samples excluded because they were collected after an on-study recurrence. (**b**) Subjects who experienced an on-study CDI recurrence were treated with antibiotics and subsequently had lower dose richness than subjects who did not experience an on-study CDI recurrence. Box plots indicating number of dose species (dose richness) observed in subject stool samples colored by whether (Y - purple) or not (N - green) they were collected following a post-treatment recurrence. Box plots used to visualize differences in dose richness show the median (central horizontal line) and interquartile range (shaded box) in each group at each time point; vertical bars indicate the most extreme non-outlier values (within 1.5 times the interquartile range), and crosses indicate outlier values (outside 1.5 times the interquartile range). (**c**) Table displaying samples numbers for B. Not all subjects who experienced an on-study recurrence provided a post recurrence sample.

One hundred and eighty-two patients were randomized in the phase 3 trial (59.9% female, mean age 65.5 years; Extended Data Table 1). All patients had a minimum of three episodes of CDI, and most (73.1%) were treated with vancomycin for the qualifying CDI recurrence. A full description of patient demographics and baseline characteristics, including sex and age, is available in Feuerstadt et al.^12^ and Cohen et al.^19^, the publications presenting clinical topline results. Patient demographics were similar in the phase 1 and phase 2 studies (Extended Data Table 1**)**. One hundred and fifty-eight baseline stool samples and 459 posttreatment sample timepoints were available for metagenomic analysis (Extended Data Fig. 1a). Baseline was defined as after antibiotic treatment for the qualifying CDI episode but prior to study treatment. More posttreatment samples were available for metagenomic analysis in the VOS-treated patients than in the placebo patients due to the significantly higher on-study CDI recurrence rate in placebo patients (that is, 12% versus 40%, respectively, by week 8 endpoint; Extended Data Fig. 1a). Some patient samples were not available due to missing specimens, protocol deviations or low sequencing read depth. Samples collected after patient exposure to posttreatment antibiotics were excluded from subsequent analysis to prevent confounding by additional antibiotic treatment, which caused a decrease in newly appearing dose species and occurred more frequently in the placebo arm (Extended Data Fig. 1b,c). Additionally, metagenomics data from 273 samples from 28 patients in the phase 1b trial (66.7% female, mean age 61.9 years) and 79 patients in the phase 2 trial (67.4% female, mean age 64.5 years) collected at timepoints shared with the phase 3 trial prior to on-study CDI recurrences (if applicable) were available for cross-study comparisons^13^ (Supplementary Table 1).

### The pharmacokinetics of VOS treatment across dosing regimens in three clinical trials

**Fig. 1:**
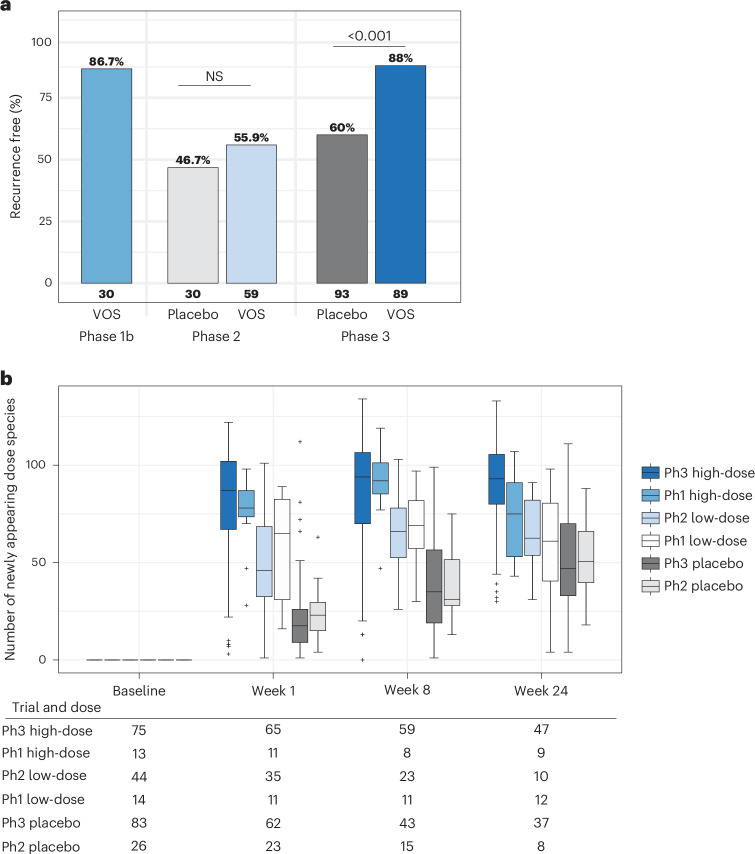
Clinical results and engraftment of dose species in patients receiving VOS treatment across trials. **a**, The percentage of patients who were recurrence free after 8 weeks is shown for the VOS (formerly SER-109) phase 1, phase 2 and phase 3 studies. Patient numbers are noted for each group. **b**, Box plots displaying VOS dose species engraftment quantified as newly appearing dose species, observed across the phase 1−3 (Ph1−3) clinical trials with sample numbers listed in the table below. VOS doses used across the three trials were categorized into high-dose regimens, encompassing the phase 3 study and higher dosing levels in the phase 1 dose-escalating study, and low-dose regimens, encompassing the phase 2 study and lower dosing levels in the phase 1 dose-escalating study (Methods). Box plots show the median (central horizontal line) and interquartile range (shaded box) in each group at each timepoint; vertical bars indicate the most extreme non-outlier values (within 1.5 times the interquartile range); and crosses (+) indicate outlier values (outside 1.5 times the interquartile range). NS, not significant.

Dosing in the phase 1 and phase 2 studies was categorized as ‘high-dose’ and ‘low-dose’ regimens as previously reported^13^. The clinical outcomes and variable levels of engraftment observed across the two trials led to the conclusion that the dose regimen used in the phase 2 trial was suboptimal compared to the higher dose regimen used in the dose-ranging arm of the phase 1b study (Fig. 1a). Based on these data, we hypothesized that delivery of a higher dose of VOS over 3 days in the phase 3 trial would result in improved engraftment, leading to a significant reduction in CDI recurrences^13^. Engraftment for each patient is defined as newly appearing VOS dose species: the number of dose species observed after treatment but absent from the patient’s baseline sample. Notably, engraftment observed in the phase 3 trial was similar to the high-dose group in the phase 1 study (Fig. 1b and Extended Data Table 2). Engraftment in the phase 3 trial was also higher than that observed in patients who received the fixed low-dose regimen in the phase 2 study at all timepoints shared (Extended Data Table 2; two-sided Mann−Whitney *U*-test (MWU), *P* < 0.01). Furthermore, engraftment in the phase 3 study was rapid and durable, as evidenced by the significantly greater number of newly appearing dose species in patients receiving VOS compared to placebo immediately after dosing that was maintained through week 24 (Fig. 1b; MWU, *P* < 0.001). In placebo patients, some newly appearing dose species were anticipated after antibiotic discontinuation because dose species are commensals found in healthy populations. However, the number of newly appearing dose species in the placebo arm was significantly lower than in the VOS arm immediately after dosing through week 24 and increased slowly over the course of the study, reflecting a gradual recovery of spore-forming Firmicutes in the absence of VOS. For the remainder of this paper, we focus on an in-depth analysis of the pharmacokinetics and pharmacodynamics of VOS in the phase 3 trial to better understand the mechanistic underpinnings of VOS efficacy in patients with rCDI.

### VOS engraftment was similar between subgroups stratified by age and antibiotic

**Extended Data Fig. 2:**
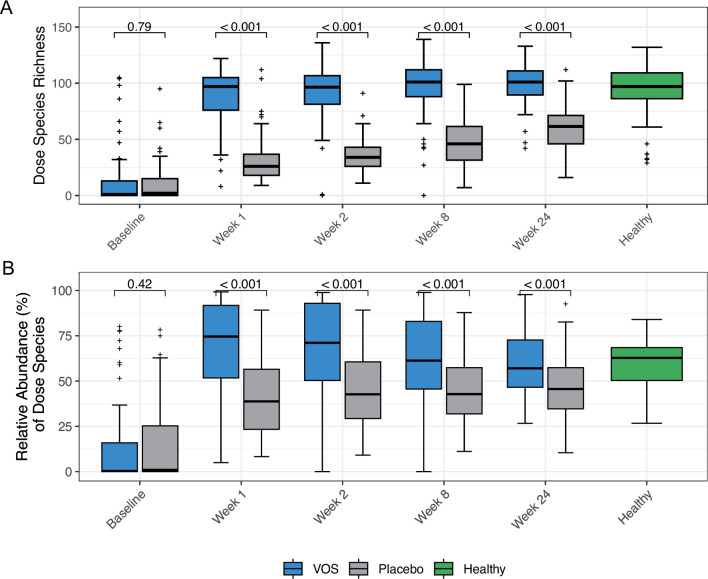
Dose species richness and abundance across study timepoints compared to healthy in Phase 3 trial. (**a**) Box plots depict dose species richness. VOS arm was not significantly different than the healthy cohort at any timepoint (p > 0.05, two-sided Mann-Whitney U-tests) whereas placebo was consistently significantly lower than healthy (p < 0.001). (**b**) Box plots depicting the summed percentages of dose species. Compared to the healthy cohort, VOS subjects had a significantly higher relative abundance of dose species at week 1 and 2 (p < 0.01, two-sided Mann-Whitney U-tests) but not at week 8 or 24 (p > 0.05). Placebo dose species abundance was significantly lower than the healthy cohort, across all post-dosing timepoints (p < 0.001). All box plots visualizing dose richness and abundances show the median (central horizontal line) and interquartile range (shaded box) in each group at each time point; vertical bars indicate the most extreme non-outlier values (within 1.5 times the interquartile range), and crosses indicate outlier values (outside 1.5 times the interquartile range). Sample sizes (VOS, PBO): Baseline (75,83); Week 1 (71,66), Week 2 (65, 55), Week 8 (64, 46), Week 24 (51, 40). Healthy cohort (68).

We compared dose species richness (the number of dose species) in phase 3 trial patients to that of healthy individuals. The distribution of dose species richness in VOS patients at week 1 through week 24 overlapped with, and was not statistically different from, the healthy cohort (Extended Data Fig. 2a; MWU, *P* = 0.2). By contrast, in the placebo arm, it was significantly lower and largely non-overlapping with the healthy cohort through week 24 (Extended Data Fig. 2a; MWU, *P* < 0.001).

**Fig. 2:**
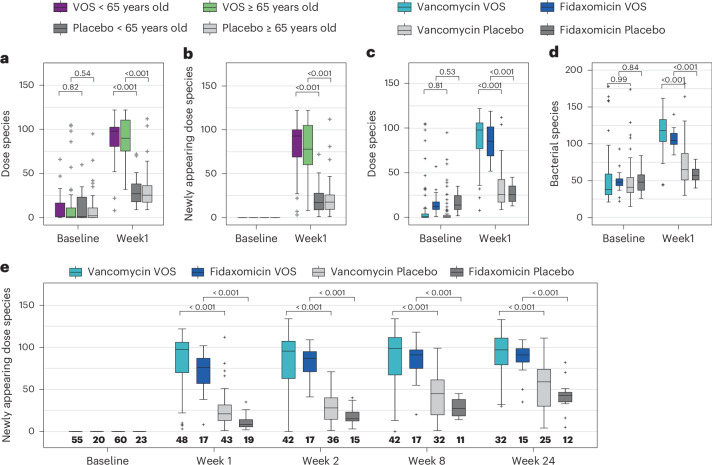
VOS dose species richness and engraftment stratified by age or antibiotic in the phase 3 trial. Box plots display dose richness (**a**) (the number of dose species) and the number of engrafting species (defined as newly appearing dose species) (**b**) observed at baseline (post-antibiotics, pre-dosing) and 1 week after treatment, in patients younger than 65 years versus patients 65 years or older. Sample sizes for dose richness (**a**) are 31, 44, 36, 47 and 32, 39, 30, 36 for VOS < 65 years, VOS ≥ 65 years, placebo <65 years and placebo ≥65 years, at baseline and at week 1, respectively. Sample sizes for newly appearing dose species (**b**) are 31, 44, 36, 47 and 27, 38, 28, 34 for VOS < 65 years, VOS ≥ 65 years, placebo <65 years and placebo ≥65 years, at baseline and at week 1, respectively. Dose richness (**c**) and species richness (**d**) observed in patients who received vancomycin versus fidaxomicin to treat their qualifying CDI episode. Sample sizes are 55, 20, 60, 23 and 53, 18, 46, 20 for VOS vancomycin, VOS fidaxomicin, placebo vancomycin and placebo fidaxomicin at baseline and at week 1, respectively. **e**, Engraftment in the antibiotic subpopulations across the entire timecourse of the phase 3 trial. Sample sizes are indicated along the *x* axis. All box plots show the median (central horizontal line) and interquartile range (shaded box); vertical bars indicate the most extreme non-outlier values (within 1.5 times the interquartile range); and crosses (+) indicate outlier values (outside 1.5 times the interquartile range). *P* values indicate single comparisons with two-sided MWU. Note that newly appearing dose species require a paired-patient baseline sample, resulting in lower post-dosing sample sizes for this metric.

**Extended Data Fig. 3:**
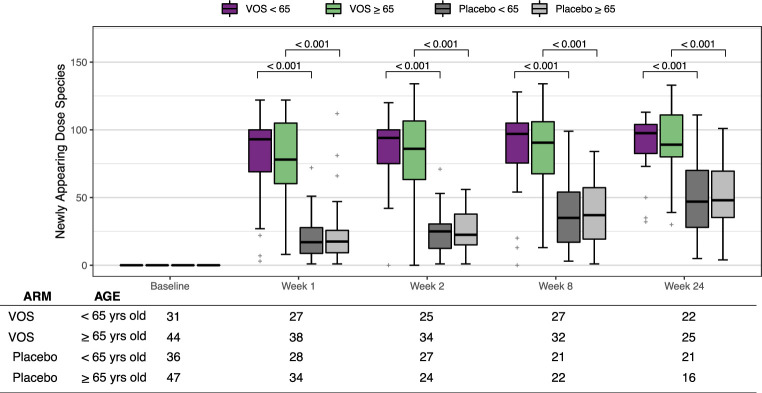
Strong and durable engraftment after VOS treatment irrespective of age in the phase 3 trial. Box plots depicting the number of newly appearing dose species (engraftment), in subjects who were less than 65 years of age or 65 years of age or older at baseline throughout the 24-week study timepoint. The number of samples analyzed for each arm and timepoint are listed in the table below the graph. All box plots show the median (central horizontal line) and interquartile range (shaded box) in each group at each time point; vertical bars indicate the most extreme non-outlier values (within 1.5 times the interquartile range), and crosses (+) indicate outlier values (outside 1.5 times the interquartile range). P-values indicate single comparisons of VOS vs placebo within each subpopulation and timepoint with two-sided Mann-Whitney U-tests.

In the VOS phase 3 trial, patients were stratified by age and by antibiotic received to treat their rCDI episode just prior to VOS or placebo treatment^12^. Dose species richness and species richness (total number of bacterial species) at baseline were low, and at baseline neither metric was significantly different between arms in patients younger than 65 years versus those 65 years or older (Fig. 2a and Supplementary Fig. 1). After dosing, in both age subgroups, newly appearing dose species were significantly greater in the VOS arm compared to the placebo arm, with this significant difference being durable through week 24 (Fig. 2b and Extended Data Fig. 3).

Richness measurements were also low at baseline compared to posttreatment samples in both vancomycin-treated and fidaxomicin-treated patients (Fig. 2c,d), although, at baseline, fidaxomicin demonstrated a slightly higher dose richness and species richness compared to vancomycin (MWU, pooled arms vancomycin versus fidaxomicin *P* values: 0.001 and 0.07, respectively). Regardless of which antibiotic was administered, the dose species richness significantly and rapidly increased from baseline after VOS treatment, resulting in markedly higher engraftment in the VOS arm compared to the placebo arm; this significant difference was durable through week 24 (Fig. 2c,e).

### VOS engraftment confirmed using strain-based methods

**Extended Data Fig. 4:**
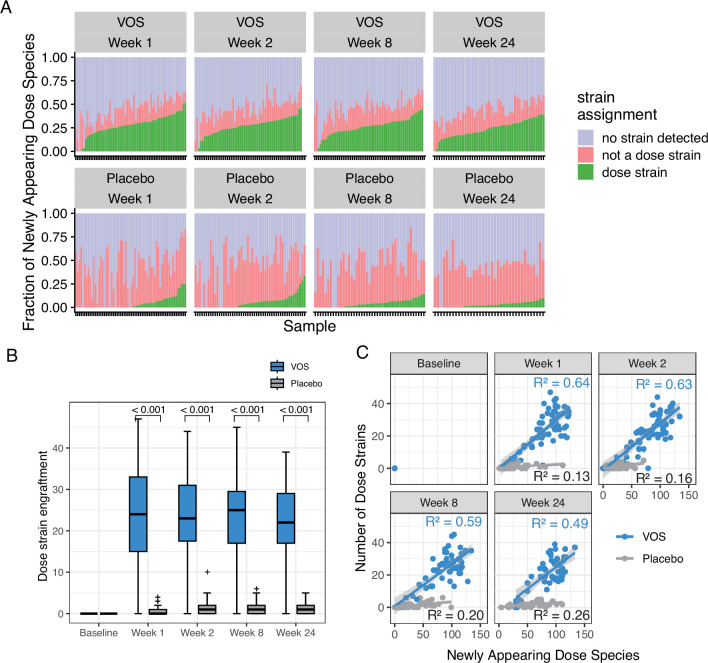
Durable strain engraftment associates with species-level engraftment in the phase 3 trial. (**a**) Fraction of the newly appearing dose species observed in each sample (see Fig. 1), that when analyzed with a strain-based method (StrainPhlAn) either matched a dose strain (dose strain - green), were phylogenetically distinct from dose strains (not a dose strain - pink) or were not detected in either the dose material or stool sample (no strain detected - lavender). Each vertical bar represents a single sample. Samples are sorted by fraction of dose strains. (**b**) Box plots indicating the number of newly appearing dose species confirmed to be dose strains using the StrainPhlAn algorithm. Box plots show the median (central horizontal line) and interquartile range (shaded box) in each group at each time point; vertical bars indicate the most extreme non-outlier values (within 1.5 times the interquartile range), and crosses (+) indicate outlier values (outside 1.5 times the interquartile range). P-values indicate single comparisons of VOS vs placebo within each timepoint using two-sided Mann-Whitney U-tests. **c**) Number of newly appearing dose species (x-axis) compared to the number of engrafting species which match dose strains (Dose Strains, y-axis). R^2^ value of linear regressions modeling dose strains by newly appearing dose species for VOS and placebo samples are written in the top and bottom of plots, respectively. Shaded regions define 95% confidence interval of the model. Sample sizes (VOS, PBO): Baseline (75,83); Week 1 (71,66), Week 2 (65, 55), Week 8 (64, 46), Week 24 (51, 40).

To evaluate the specificity of the species-based assessment of VOS engraftment, we used StrainPhlAn to classify newly appearing dose species in each patient as either a dose or a non-dose strain. A strain designation could not be assigned to approximately half of newly appearing dose species (Extended Data Fig. 4). Regardless of the reduced sensitivity, the strain-based approach showed significantly greater numbers of dose strains in the VOS arm compared to the placebo arm (Extended Data Fig. 4; MWU, *P* < 0.001). Additionally, strain-based and species-based engraftment measures correlated with the relationship being stronger in the VOS arm (Extended Data Fig. 4c; *R*^2^ values at week 1: VOS: 0.64, placebo: 0.13).

### VOS treatment is associated with broad compositional changes more closely resembling gastrointestinal microbiomes from healthy individuals

**Fig. 3:**
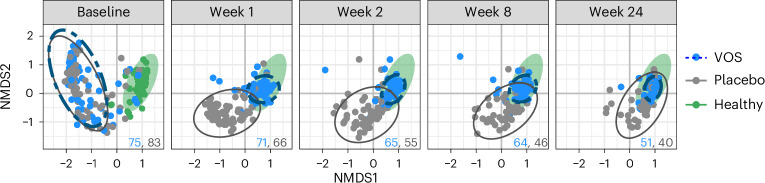
Compositional distances between patient microbiomes in the phase 3 trial. NMDS plot displaying Bray−Curtis dissimilarity of microbial composition between patient samples from baseline (before treatment) through 24 weeks after treatment in the phase 3 study (*k* = 3, stress = 0.13), including ellipses outlining 95% confidence intervals of the bivariate normal fit to each group. A cohort of 68 healthy individuals is also plotted as a reference at the pretreatment baseline (green points) with healthy population data ellipse (green filled) projected across all timepoints. VOS (blue) and placebo (gray) sample sizes are shown in the bottom right corner, respectively. NMDS axis 3 is provided in Supplementary Fig. 2.

We next evaluated compositional distances between bacterial communities over time, with a healthy cohort serving as a reference. We found that baseline samples in non-metric multidimensional scaling (NMDS) projections clustered away from healthy cohort samples (Fig. 3) and clustered by standard-of-care antibiotic (Supplementary Fig. 3). After treatment with VOS, patient samples rapidly transitioned away from the baseline cluster toward the healthy cohort (Fig. 3; week 1) and were stable thereafter (Fig. 3; weeks 2, 8 and 24). By contrast, samples from patients receiving placebo gradually transitioned away from baseline, with many remaining outside of the healthy cohort cluster through week 24. Minimal separation was observed along NMDS axis 3 (Supplementary Fig. 2). Box plots showing Bray−Curtis distances, confirming these trends, are displayed in Supplementary Figs. 4–6. PERMANOVA and dispersion tests comparing treatment arms within each timepoint differ significantly in both the location of samples and the size of clusters (Fig. 3; *P* < 0.001).

**Fig. 4:**
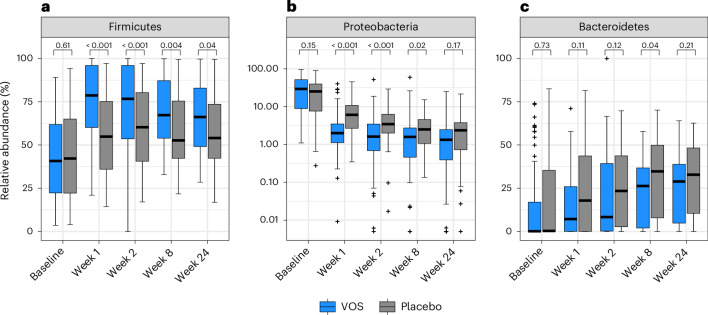
Relative abundances of the three major gastrointestinal bacterial phyla across the phase 3 study. Box plots depict relative abundances of Firmicutes (**a**), Proteobacteria (**b**) and Bacteroidetes (**c**) phyla. Note that linear scale is used for Firmicutes (**a**) and Bacteroidetes (**c**). By contrast, Proteobacteria (**b**) is typically only a minor component of the gastrointestinal microbiome in healthy individuals and, thus, is shown using a log scale to better view the distribution of sample abundances below 1%. All box plots show the median (central horizontal line) and interquartile range (shaded box) in each group at each timepoint; vertical bars indicate the most extreme non-outlier values (within 1.5 times the interquartile range); and crosses (+) indicate outlier values (outside 1.5 times the interquartile range). *P* values indicate single comparisons between treatment arms at each timepoint with two-sided MWU. Sample sizes (VOS, placebo): baseline (75, 83); week 1 (71, 66); week 2 (65, 55); week 8 (64, 46); week 24 (51, 40).

To assess the taxonomic changes underlying these broad shifts, we first evaluated changes in abundance of dominant gastrointestinal-associated phyla. Consistent with engraftment of drug product species, we observed a jump in the relative abundance of Firmicutes (summed dose and non-dose species) (Fig. 4a) and in the summed relative abundance of dose species in VOS patients after treatment (Extended Data Fig. 2b). Dose species abundances in the VOS arm after treatment were initially higher than abundances observed in the healthy cohort (MWU, *P* < 0.001) but declined over time, reaching similar values to the healthy cohort by week 8 (Extended Data Fig. 2b; MWU, *P* = 0.27). There was a reciprocal rapid decline in the relative abundance of the Proteobacteria from baseline to week 1 in VOS patients (Fig. 4b). Similar dynamics were observed in the placebo arm, although the changes in Firmicutes and Proteobacteria were more gradual, with Firmicutes not approaching similar abundances as the VOS arm until week 24 (Fig. 4a,b; VOS versus placebo, MWU, *P* < 0.05 through week 8). In both arms, the Bacteroidetes phylum gradually increased in relative abundance through 24 weeks (Fig. 4c; VOS versus placebo, MWU, *P* > 0.05 at weeks 1, 2 and 24).

**Fig. 5:**
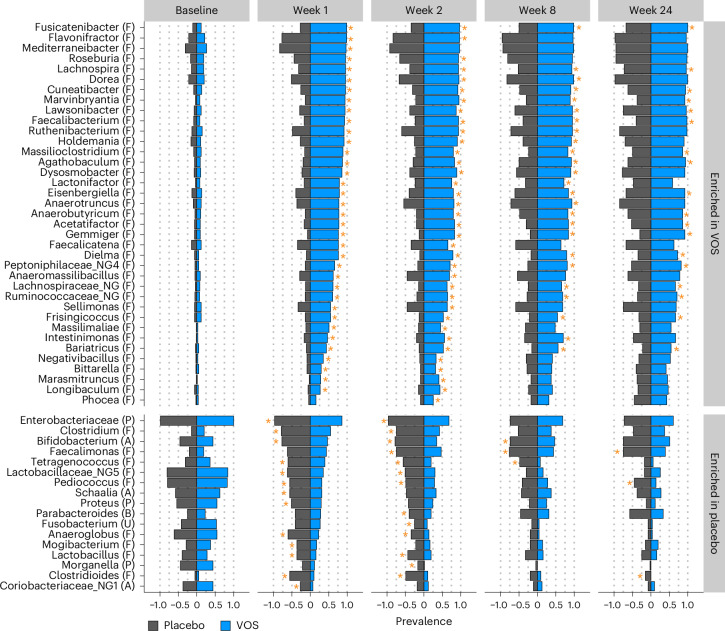
Prevalence of bacterial genera within each treatment arm in the phase 3 study. Letters in parentheses next to group names indicate phylum membership: (F), Firmicutes; (P), Proteobacteria; (A), Actinobacteria; (U), Fusobacteria; (B), Bacteroidetes. Genera identified in at least 30% of samples and with significant differences in prevalence between treatment arms for at least one timepoint are shown. Significant differences in prevalence between treatment arms are designated with gold stars (Fisher’s exact test, FDR < 0.05). Sample sizes (VOS, placebo): baseline (75, 83); week 1 (71, 66); week 2 (65, 55); week 8 (64, 46); week 24 (51, 40).

When comparing compositional differences between VOS and placebo at finer taxonomic resolution, only Firmicutes genera were found to be more prevalent in VOS patients (Fig. 5). Conversely, the Gram-negative *Enterobacteriaceae* family (for example, *Klebsiella sp*. and *Escherichia coli*) was more prevalent in placebo-treated patients (Fig. 5 and Supplementary Fig. 7). Also, we observed higher prevalence or abundance of species associated with pathogenesis or disease in the placebo arm (for example, *Fusobacterium nucleatum*^20^, *Enterococcus* spp.^21^and *Klebsiella* spp.^22^). Consistent with the clinical success of VOS, we observe significantly less *C. difficile* in the VOS arm relative to the placebo arm (Fig. 5 and Supplementary Fig. 7).

### VOS treatment leads to changes in metabolites relevant to the *C. difficile* life cycle

**Fig. 6:**
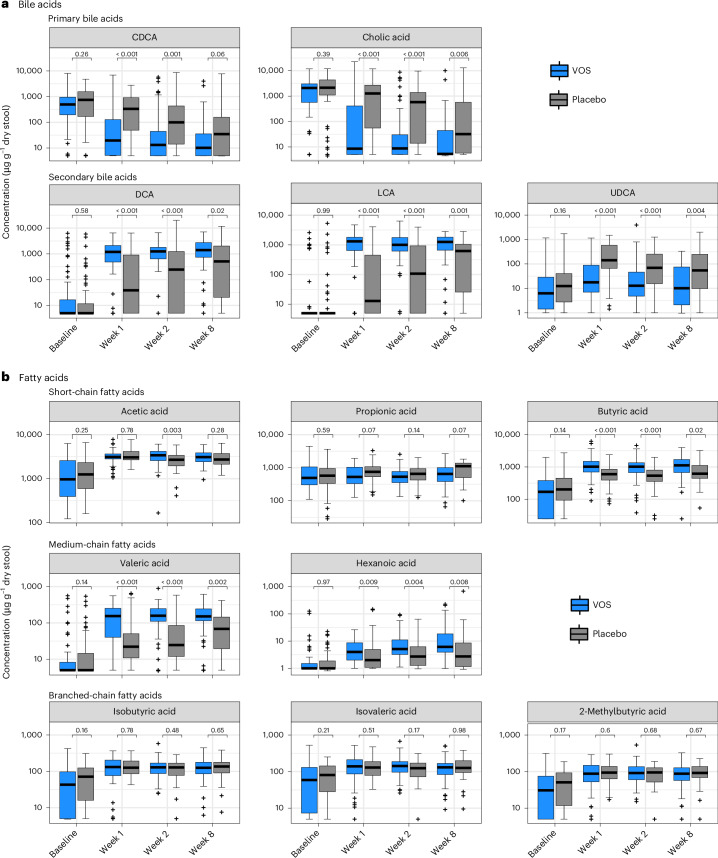
Comparison of bile acids and fatty acids across treatment arms from baseline through week 8. Box plots depict median concentration of various primary and secondary bile acids (**a**) and short-chain, medium-chain and branched-chain fatty acids (**b**) in patient stool samples. All box plots show the median (central horizontal line) and interquartile range (shaded box) in each group at each timepoint; vertical bars indicate the most extreme non-outlier values (within 1.5 times the interquartile range); and crosses (+) indicate outlier values (outside 1.5 times the interquartile range). *P* values indicate single comparisons with two-sided MWU. Sample numbers for bile acids are 77, 72, 66 and 63 for VOS and 81, 66, 56 and 45 for placebo, across timepoints, respectively. Sample numbers for fatty acids are 69, 63, 59 and 56 for VOS and 67, 53, 47 and 36 for placebo, across timepoints, respectively.

Targeted metabolomics showed elevated primary bile acids and depressed secondary bile acids in both treatment arms at baseline, as observed previously in this disease state (Fig. 6a)^23^. After dosing, shifts in bile acid concentrations were rapid in the VOS arm compared to the placebo arm. Specifically, concentrations of the primary bile acids cholic acid and chenodeoxycholic acid (CDCA) were highest at baseline and remained elevated in the placebo arm relative to the VOS arm through week 2 after dosing (Fig. 6a; MWU, *P* ≤ 0.001). In parallel, concentrations of secondary bile acids deoxycholic acid (DCA) and lithocholic acid (LCA) were low at baseline and increased rapidly in the VOS arm, leading to greater concentrations compared to placebo through week 8 (MWU, *P* ≤ 0.02). By contrast, ursodeoxycholic acid (UDCA) concentrations were lower in the VOS arm than in the placebo arm (MWU, *P* < 0.01).

**Extended Data Fig. 5:**
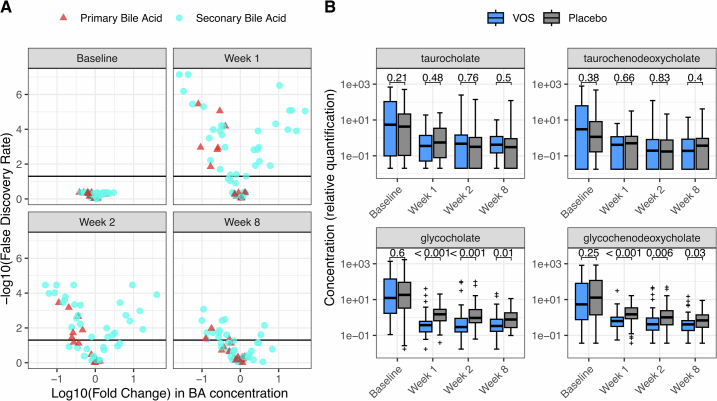
Enrichment of primary and secondary bile acids in phase 3 subject stool samples. (**a**) Volcano plot showing enrichment of primary and secondary bile acids in the VOS (Log10(Fold Changes) > 0) versus the placebo arm (Log10(Fold Change) < 0) in a global metabolomics assay across time points. The x-axis represents bile acid concentration differences, while the y-axis shows statistical significance between arms (black horizontal line, FDR < 0.05). **b**) Comparison of primary conjugated bile acid concentrations observed in VOS and placebo arms across timepoints. Box plots show the median (central horizontal line) and interquartile range (shaded box) in each group at each time point; vertical bars indicate the most extreme non-outlier values (within 1.5 times the interquartile range), and crosses (+) indicate outlier values (outside 1.5 times the interquartile range). P-values indicate single comparisons with two-sided Mann-Whitney U-tests. Sample numbers across timepoints for VOS and placebo are 39, 39, 53 and 29 and placebo are 21, 25, 15 and 14, respectively.

We expanded upon this analysis by evaluating the change in a broader set of bile acids detected using global metabolomics. In agreement with the targeted assay, both cholic acid and CDCA were significantly reduced by treatment with VOS. Additionally, primary bile acids glycocholic acid, glycochenodeoxycholic acid, glycochenodeoxycholic acid 3-sulfate, cholic acid sulfate and chenodeoxycholic acid sulfate (2) were significantly reduced in VOS-treated patients after dosing, and diverse sets of secondary bile acids were enriched in the VOS and placebo arms (Extended Data Fig. 5 and Supplementary Table 2).

A targeted fatty acid metabolomics assay revealed that whereas concentrations of acetate and propionate were similar between arms, the longer C-backbone short-chain and medium-chain fatty acids butyrate, valerate and hexanoate increased rapidly in the VOS arm, leading to greater concentrations compared to placebo through week 8 (Fig. 6b; MWU, *P* < 0.05). The branched-chain fatty acids isobutyrate, isovalerate and 2-methylbutyrate increased from baseline but did not differ significantly between arms.

**Extended Data Fig. 6:**
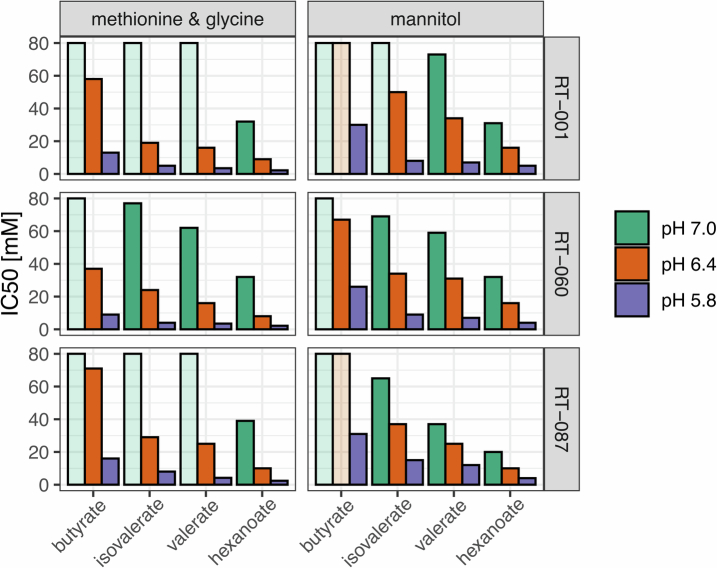
Concentration dependent fatty acid growth inhibition. The half maximum inhibitory concentration (IC_50_ [mM]) of butyrate, isovalerate, valerate and hexanoate that results in 50% reduction of OD_600_ (yield) in 24 hour batch cultures of the *C. difficile* ribotypes (Ribotypes: R-001, ATCC 9689; R-069, ATCC 043593; R-087, ATCC 43255) in medium with mannitol or methionine & glycine at indicated pH values. Transparent bars represent IC_50_ values > 80 mM, the highest concentration of fatty acid tested. Each bar represents the average of triplicate cultures.

Although the impact of bile acids on *C. difficile* germination and growth is well established in the literature^4,5,24^, evidence establishing the impact of different short-chain fatty acids and medium-chain fatty acids on *C. difficile* growth is more limited^8,10,25–28^. To better understand the impact of these metabolites on *C. difficile* growth, we evaluated the impact of different concentrations of butyrate, isovalerate, valerate and hexanoate in vitro. All tested fatty acids were able to slow down or inhibit in vitro growth of *C. difficile* (Extended Data Fig. 6). Although inhibition was influenced by growth substrate across media types, inhibition increased with fatty acid concentration, acidity of the medium and C-backbone length of the fatty acid. Overall patterns of inhibition were consistent across the three *C. difficile* ribotypes tested.

### Stool-derived spore fractions produce metabolites inhibitory to *C. difficile* growth in vitro

**Extended Data Fig. 7:**
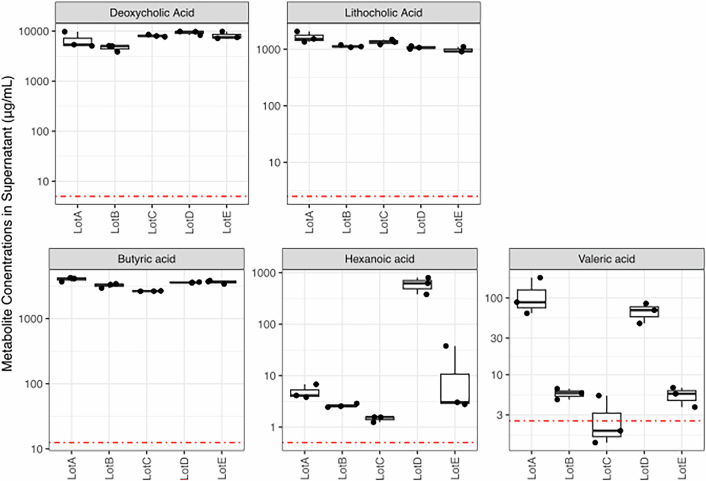
Metabolite concentrations in supernatant of dose material grown *in-vitro.* Cultures were run in triplicate, with black circles indicating individual values for each donor sample. Red dotted line indicates the lowest concentration in the calibration standard range (below lower limit of quantification), values below which were extrapolated. Media control is un-inoculated media samples included in growth plates. All box plots show the median (central horizontal line) and interquartile range (shaded box) in each group at each time point; vertical bars indicate the most extreme non-outlier values (within 1.5 times the interquartile range), and crosses (+) indicate outlier values (outside 1.5 times the interquartile range).

**Extended Data Fig. 8:**
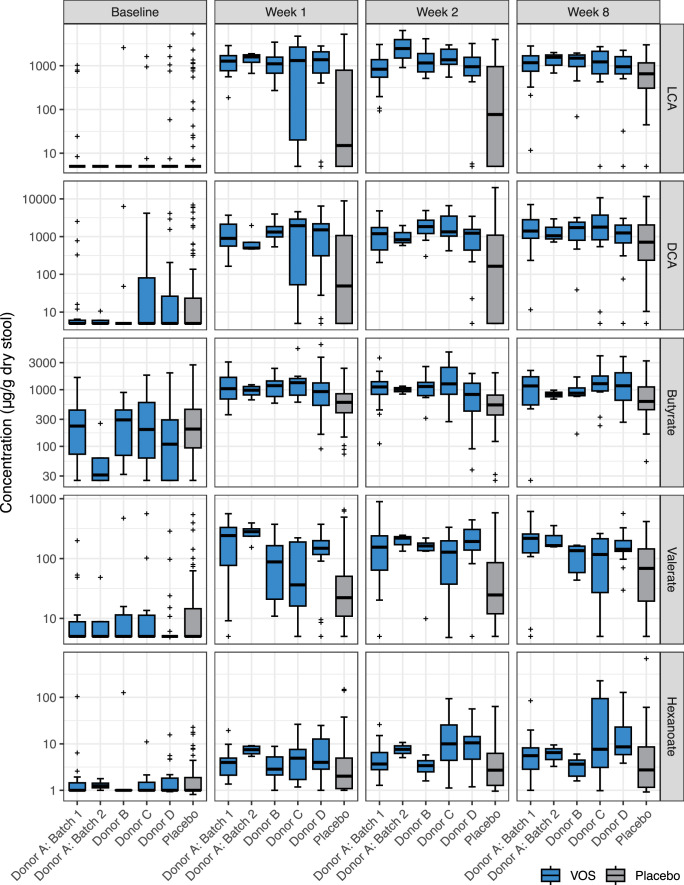
Metabolite concentrations in Phase 3 subject stool samples, plotted by donor batch received. Box plots indicate concentrations of various fatty acid metabolites in subject stool samples plotted by which donor batch was used to generate the VOS treatment they received. All box plots show the median (central horizontal line) and interquartile range (shaded box) in each group at each time point; vertical bars indicate the most extreme non-outlier values (within 1.5 times the interquartile range), and crosses (+) indicate outlier values (outside 1.5 times the interquartile range). Number of subjects with metabolite data that received each batch are as follows: Donor A Batch 1: 25, Donor A Batch 2: 4, Donor B: 12, Donor C: 14, Donor D: 29, Placebo: 87.

To further assess the observed changes in metabolites to dosing with VOS, we examined the production of DCA, LCA, butyrate, valerate and hexanoate in cultured dose material from the four donors used in the VOS phase 3 trial. Donor A contributed to two dose batches, both of which were assayed. Because in vitro culture conditions do not reflect the complex environment of the gastrointestinal tract (for example, single medium, small volumes and no host interaction), we restricted our analysis to binary outcomes only—production versus non-production of metabolites as defined by detection of metabolites in culture supernatant above those of the uninoculated medium controls and above the lower limit of assay calibration curves in at least one of three replicate cultures. All five batches displayed the anticipated bile acid and fatty acid metabolic activities (Extended Data Fig. 7). Parsing phase 3 patient metabolites by batch of VOS received showed that the median concentration of each metabolite was consistently higher across VOS batches compared to the placebo group, although low and imbalanced numbers between batches limited the power of statistical tests (Extended Data Fig. 8).

## Discussion

VOS, manufactured by purifying Firmicutes spores from donor material with a greater than 200-fold reduction in non-spore stool matter, is composed of only a small fraction of the microbial taxa found in full-spectrum FMT products, making it a unique product^13^. To our knowledge, VOS is the only live, orally delivered microbiome therapeutic to date to meet its primary efficacy endpoint in a phase 3 randomized controlled trial: to significantly reduce rCDI compared to placebo^12^. Estimates of efficacy for microbiome therapeutics and FMTs in rCDI vary from 44% to 91% based on the specific therapeutic agent, route of administration, trial design and diagnostic test used^29^. VOS achieved 88% efficacy for prevention of recurrence compared to 60% of placebo and a number needed to treat of four^12^. In this analysis of the impact of VOS on the gastrointestinal microbiome, we demonstrate the importance of informing clinical development of microbiome therapeutics by evaluation of drug pharmacokinetics and pharmacodynamics.

Engraftment of drug species is a critical step in driving the pharmacodynamic changes needed to prevent rCDI. Analyses across the VOS clinical development program confirm our previous hypothesis^13^: in the phase 2 study, VOS dosing was inadequate to drive sufficient engraftment and subsequent rCDI prevention. Optimization of dosing in the phase 3 trial led to a significant increase in engrafting species, leading to a significant reduction in risk of CDI recurrence^12^.

Engraftment of dose species in patients treated with standard-of-care antibiotics followed by VOS was rapid and remained elevated through 24 weeks of follow-up. By contrast, recovery of commensal spore-forming bacteria was delayed in patients who received standard-of-care antibiotics followed by placebo and never reached the same level as VOS. This gradual increase of spore-forming Firmicutes in placebo is consistent with the expectation that microbiome diversity rebounds after discontinuation of antibiotics. The placebo control arm is key to delineating the extent to which VOS dosing increased Firmicute species after release from antibiotics^30–33^. The speed of recovery is key to clinical outcome, as demonstrated by the rapid recurrence of CDI that was observed within 4 weeks after antibiotic discontinuation in the placebo arm of the phase 3 trial. Collectively, these data across VOS clinical development suggest that the pace and magnitude of VOS engraftment and microbiome changes are likely critical to end the cycle of CDI recurrence^12,19^.

We further evaluated engraftment using the strain-based analytical tool StrainPhlAn^34,35^, which reinforced the species-based engraftment conclusions. Strain-based analyses have higher specificity than species-based approaches; however, strain-based approaches suffer from lower sensitivity because distinguishing strains requires higher sequencing depth of the organism in both dose and patient samples. Patient samples and VOS contain spore-forming Firmicutes that vary in abundance, resulting in a substantial portion of species for which no strain assignment can be made. Given that we found concordance between the approaches, and that the phase 2 analysis and subsequent phase 3 design were based on species, we focused on the higher-sensitivity, species-based approach, with the placebo arm providing a benchmark for the number of species that recover without intervention. The modest and gradual increases in both dose species and strains observed in the placebo arm after antibiotic discontinuation reinforce the importance of a placebo arm when assessing microbiome therapeutics.

We showed in a previous clinical trial that microbiome conditioning with antibiotic is important for engraftment of spore-based products^36^. Data from a phase 2 trial of a designed consortium suggested that residual antibiotics may have inhibited early engraftment^37^, and FMT meta-analyses found that antibiotic and lavage pretreatment are among the strongest predictors of engraftment^38,39^. VOS engraftment may have been facilitated by the laxative taken between completion of antibiotic and start of VOS treatment. The rationale is that laxative rapidly reduces levels of residual antibiotics in the gastrointestinal tract, enabling VOS treatment 2−4 days after antibiotics without antibiotic interference and, thereby, allowing VOS species to colonize before significant competition from rebounding native species^40^. Note that placebo patients also took a laxative before dosing.

VOS led to greater clinical efficacy compared to placebo for patients stratified by age (<65 years or ≥65 years) and antibiotic^12^. Consistent with these results, we observed a rapid increase in newly appearing dose species across all subgroups. Age is a well-documented risk factor for CDI recurrence and may be related to the senescence of the microbiome in older populations^41^; however, in this study, engraftment differences based on age were not observed. Similarly, treatment with fidaxomicin is hypothesized to be less disruptive to the microbiome compared to vancomycin^42^. However, study patients had only modest baseline differences in Firmicutes richness, which were dwarfed by the increased dose species after dosing with VOS compared to placebo. Any theoretical advantage of fidaxomicin over vancomycin was not supported by the high recurrence rates observed in placebo patients after either antibiotic^12^. Collectively, these data suggest that treatment with a Firmicutes spore product is efficacious for prevention of rCDI independent of age and antibiotic.

To assess the pharmacodynamic impact of VOS, we first evaluated the impact of dosing on the entire microbiome community. The relative abundances of Firmicutes and Bacteroidetes in the gastrointestinal tract vary widely across healthy populations, whereas Proteobacteria consistently represent a small fraction, ranging from less than 1% to approximately 2%^40,43–45^. However, after antibiotic treatment for rCDI, the gastrointestinal microbiome is characterized by enrichment of Gram-negative Proteobacteria and depletion of Firmicutes and Bacteroidetes. The rapid gains in the richness and abundance of spore-forming Firmicutes observed after dosing with VOS were accompanied by a reciprocal loss of Proteobacteria. These changes resulted in a microbiome profile much more similar to healthy individuals within 1 week of initiating VOS dosing compared to the placebo arm. After completion of antibiotics, the relative abundance of Bacteroidetes increased gradually in both arms, reinforcing that treatment with spore-forming Firmicutes alone is sufficient to reduce the risk of CDI recurrence.

The pharmacodynamics of VOS were further assessed by measuring the substrate and products of primary to secondary bile acid conversion. These metabolites play important roles in *C. difficile* growth inhibition and are carried out by a limited number of Firmicutes groups, including *Ruminococcaceae*, *Lachnospiraceae* and *Peptostreptococcaceae*^46^, members of which comprise VOS and, therefore, provide a targeted measure of pharmacodynamics. By contrast, primary bile acid deconjugation, the first microbially driven step in bile acid conversion carried out by bile salt hydrolase (BSH) enzymes, is widely distributed^47^, including in Bacteroidetes and some Proteobacteria, and, therefore, is not expected to be a precise measure of pharmacodynamics and a spore-forming Firmicute product. Consistent with this, we observed similar reductions in taurocholic acid across both arms, suggesting that, although BSH activity reducing this potent *C. difficile* germinant may be necessary, it is not sufficient to explain VOS efficacy. Notably, glycocholic acid was significantly reduced in VOS recipients, consistent with proposed potential differences in the deconjugation dynamics of these two bile acids^48^.

In the phase 2 study, stronger engraftment was associated with clinical outcome and was well correlated with the abundance of secondary bile acids LCA and DCA^13^. In both the phase 2 and phase 3 studies, treatment with VOS was associated with significantly increased concentrations of LCA and DCA and a reciprocal decline of primary bile acids cholic acid and CDCA, germinants of *C. difficile* spores. UDCA concentrations were lower in VOS patients compared to placebo patients, consistent with deconjugated primary bile acids being directed toward 7α-dehydroxylation and away from the UDCA pathway. VE303, a microbiome therapeutic currently being evaluated for rCDI prevention in a phase 3 trial, directs bile acids through the UDCA pathway^49^. Results of the VE303 phase 3 trial will shed light on whether this alternative strategy also leads to an effective rCDI treatment^10^.

Previous studies linked fatty acids to inhibition of *C. difficile*^8,25–28^, but the reported effects varied among studies, and the precise contribution was confounded by effects of pH, fatty acid concentrations, growth medium and *C. difficile* strain variability. In the present study, we systematically evaluated the impact of increasing concentrations of butyrate, isovalerate, valerate and hexanoate in vitro, buffering media at pH values typical of the proximal, mid and distal colon. Because bacterial sensitivity to environmental stressors depends on different metabolic pathways, we evaluated two growth substrates, a sugar alcohol and an amino acid mix, both used by *C. difficile*in vivo and metabolized by different pathways^50–52^. All tested fatty acids were able to inhibit growth of *C. difficile*, with potency increasing with C-backbone length. Notably, in the phase 3 clinical study, hexanoate as well as valerate and butyrate concentrations were low at baseline and increased significantly after VOS treatment. Taken together, these results support the contribution of fatty acids to inhibiting *C. difficile*^25–28^. Fatty acids have also been shown to suppress colonic inflammation and support epithelial barrier integrity, which may facilitate return to gastrointestinal homeostasis after CDI^9,10^.

Compared to traditional drugs that may target a single metabolite or enzyme, VOS introduces live bacteria capable of performing multiple functions and, thereby, durably impacts a myriad of physiological processes. Generation of secondary bile acids and SCFAs by dose material in in vitro cultures provides strong evidence that VOS bacteria directly modulate these two distinct metabolite classes. In combination with the phase 3 clinical results^12,13,53^, these pharmacodynamic findings support the role of bile acids and SCFAs in preventing CDI recurrence.

The greatest strength of these post hoc microbiome analyses for supporting an understanding of mechanisms of action is that they come from a double-blind, placebo-controlled trial in patients with toxin-proven disease. Additionally, to our knowledge, VOS is the only rCDI microbiome therapeutic or rCDI investigational microbiome therapeutic to have demonstrated statistically significant changes in metabolite concentrations compared to placebo. Other recently published studies reporting on rCDI microbiome therapeutics or rCDI investigational microbiome therapeutics have not included placebo data^49,54^, divided the data between by clinical outcome on the study rather than by treatment group^15,55^ or did not report statistically significant differences between arms^55^.

Limitations of this study include the limited number of CDI recurrences in the VOS arm, preventing examination of associations between engraftment or metabolites and clinical outcome. Additionally, metagenomic sequencing provides relative abundance measures, so it is unclear if taxa were decreasing or increasing in absolute abundance in the gastrointestinal tract. However, this is an inherent limitation of DNA sequencing methods used in the field and not unique to this study. An additional limitation is the low number of donor batches used in the VOS phase 3 study.

VOS significantly reduced the risk of rCDI in a phase 3 placebo-controlled trial in patients with toxin-proven disease. Although the mechanisms of action of VOS have not been fully elucidated, these comprehensive compositional and functional assessments support the multifaceted pharmacological impact of targeted live, microbiota-based therapeutics (in this case, VOS) to replenish Firmicutes richness and abundance and drive subsequent changes in bacterial-derived metabolites, which may act synergistically to halt the recurrence cycle of CDI. These data support a potential role for VOS, after antibiotic therapy, to restore the microbe-associated metabolic functions needed to prevent CDI recurrence.

## Methods

### Study overview—ethics

The VOS phase 3 trial (ECOSPOR III) was a multicenter, randomized, double-blind, placebo-controlled study conducted at 56 US and Canadian sites from July 2017 to September 2020 (ClinicalTrials.gov: NCT03183128). The study protocol and amendments are available for download from Feuerstadt et al.^12^. The institutional review board for each study site reviewed and approved the protocol and applicable amendments, and all patients provided written informed consent at screening (Supplementary Table 3). The trial followed the CONSORT reporting guideline.

### Study overview—trial design

The VOS phase 1 trial was an open-label study conducted at three sites to evaluate the safety, dosing and efficacy of VOS for the prevention of multiply recurrent *C. difficile*, as described previously^18^. A subsequent phase 2 randomized, placebo-controlled (2:1, VOS:placebo) study evaluated a fixed dose for the prevention of multiply recurrent *C. difficile*, as described previously^13^. For the phase 3 study, eligible adults 18 years of age or older with three or more CDI episodes within 12 months, inclusive of the qualifying acute episode, were enrolled. The qualifying episode was defined as follows: (1) three or more unformed bowel movements over two consecutive days, (2) a positive *C. difficile* toxin test and (3) symptom resolution after 10−21 days of standard-of-care antibiotics.

Antibiotic treatment for the qualifying episode of rCDI was at the discretion of the treating investigator. Patients were stratified by age (<65 years or ≥65 years) and antibiotic received for the qualifying episode (vancomycin or fidaxomicin). Patients were randomly assigned 1:1 to VOS (approximately 3 × 10^7^ spore colony-forming units) or matching placebo administered as four oral capsules once daily over three consecutive days^12^. All patients, site staff and the sponsor were blinded to treatment assignment. Patients were instructed to take a laxative 1 day prior to treatment initiation to reduce residual antibiotic in the gastrointestinal tract, to minimize inactivation of this live biotherapeutic. Patients were monitored for up to 24 weeks for CDI recurrence, defined as follows: onset of three or more unformed bowel movements per day over two consecutive days, a positive *C. difficile* stool toxin assay, assessment by the investigator that antibiotic treatment was warranted and persistence of diarrhea until initiation of antibiotics.

The primary efficacy outcome measure was the rate of CDI recurrence up to 8 weeks after treatment initiation in patients who received VOS compared to patients who received placebo. Preplanned exploratory microbiome endpoints were composition of the gastrointestinal microbiome from baseline through week 24 in each treatment group and composition of bile acids in stool from baseline through week 8 after treatment in each treatment group. In this expanded post hoc analysis, we investigated engraftment of VOS spore-forming species in the context of previous VOS trials^13^. Then, we explored the impact of the engraftment in the VOS phase 3 trial (ECOSPOR III) on broader patient microbiome composition and stool metabolites (that is, bile acids and fatty acids) through week 24.

Sex was not considered in the clinical trial study design. Sex was determined based on self-reporting or health records, and sex-based analyses were connected and reported for the ECOSPOR III primary endpoint in Berenson et al.^56^. A treatment effect of lower CDI recurrence in VOS versus placebo did not differ between males and females. Therefore, sex-based microbiome analyses were not executed.

### VOS manufacturing and dose characterization

Donor screening and the manufacturing program were reviewed by the FDA^12,13,18^. Donors underwent extensive examinations, including laboratory testing before, during and after donation periods before the material was released for manufacturing. The manufacturing process uses ethanolic inactivation and other clearance steps to kill and remove non-spore microorganisms, including potential adventitious bacteria^17^ and viruses^16^. The preparation of VOS capsules relies upon filtration and centrifugation operations, which remove approximately 99% of the non-spore fecal mass, resulting in an average of less than 20 mg of residual solids per dose^13^. The increased quantity of residual solids reported here versus that reported in the phase 2 study is due to higher dosing in the phase 3 trial. VOS batches were characterized using an in vitro microbiological growth assay. Spore preparations are treated with a germinant mixture and then inoculated into a panel of 11 media conditions spanning diverse carbon sources, in quadruplicate and grown out anaerobically for 5 days. Growth assay material, as well as the original dose material, underwent genomic DNA extraction and shotgun metagenomics sequencing (MGX), and the resulting spore-forming species lists were combined to generate a single VOS dose definition.

### Sampling

Stool samples were collected before treatment (baseline within 3 days after completion of antibiotic treatment for the qualifying CDI episode) and after treatment at study weeks 1, 2, 8 and 24. Samples were collected in tubs and shipped on frozen gel packs to a central laboratory where they were homogenized, aliquoted and then immediately frozen at −80 °C. Unamended (neat) aliquots were used for bile acid and fatty acid measurements, and 12% weight/weight stool suspensions in 95% ethanol were generated for MGX.

In a previously published post hoc analysis of combined VOS phase 1 and phase 2 results, dosing levels of a phase 1 dose-ranging study were divided into ‘low-dose’ and ‘high-dose’ categories, with the phase 2 ‘fixed dose’ assigned to the low-dose category^13^. Based on these results, the phase 3 study was designed such that patients would receive the high dose. Here, metagenomics samples from the phase 1 and phase 2 studies, collected prior to on-study CDI recurrences and at shared timepoints with the phase 3 trial, were included for cross-study engraftment comparisons. For additional details and demographic information, see Khanna et al.^18^ and McGovern et al.^13^. The institutional review board for each study site reviewed and approved the protocol, and all patients provided written informed consent.

Additionally, to generate a non-CDI comparator dataset, a single stool sample was collected from each member of a cohort of self-identified healthy individuals who reported no oral, intravenous or intramuscular antibacterial exposure within the last 12 weeks and no antifungal, antiviral or antiparasitic exposure within the last 8 weeks. The cohort had an average age of 30 years and an average body mass index of 23 kg m^−2^ and was 44% female. Samples were collected and processed with the same procedure as samples from the clinical study.

### Microbiome and metabolomics profiling

For the phase 3 trial, DNA was extracted in-house from patient stool and drug materials using the Omega Mag-Bind Universal Pathogen Kit (Norcross). Libraries were prepared using Illumina DNA Flex kits and sequenced on the Illumina NovaSeq platform to a target sequence depth of 10 gigabases for patient samples at Clinical Laboratory Improvement Amendments (CLIA)/College of American Pathologists (CAP)-certified and Good Laboratory Practice (GLP)-compliant microbiome profiling facilities at Diversigen, Inc. Microbe taxonomic profiling for all MGX data used MetaPhlAn2 software^57,58^, which aligns MGX sequenced reads to species-specific markers to produce relative abundance matrices. A proprietary database of species markers was used that included taxonomic markers sourced from genomes of species in the Seres strain library as well as publicly available genomes. To monitor for contamination, each 96-well DNA extraction plate included a control sample composed of liquid media from a pure bacterial culture. After DNA extraction, a second control composed of bacterial DNA was added to a well in each sequencing plate. Both controls were sequenced alongside study samples, and no unexpected organisms were detected.

For species-level taxonomic profiling of patient stool, MGX reads in the phase 3 trial were first mapped to species-specific markers, to determine the number of mappable reads in each sample. To ensure robust comparisons of species numbers across samples and trial datasets, the number of reads mapping to species markers was downsampled to 163,000 reads (randomly selected) for each sample. This number of reads was chosen to optimize sequencing depth in individual samples while maintaining the total number of samples included in the phase 3 dataset and was applied to data from all three trials. Samples with fewer than 163,000 mappable reads were excluded from analyses (Extended Data Fig. 1).

To determine if the newly appearing dose species yield similar or different engraftment trends to strain-level engraftment, StrainPhlAn (version 4 with default parameters) was applied to combined patient and dose material MGX datasets. A cutoff of 0.02 or smaller normalized phylogenetic distance, calculated from StrainPhlan phylogenetic trees, was used to define same strains. This threshold represents the 10% quantile of the distribution of normalized phylogenetic distances between the independent healthy cohort and dose material, where strain overlap is anticipated to be minimal. Quantitative assessment of changes in bile acid and fatty acid concentrations was conducted on homogenized, lyophilized stool samples, spiked with internal standards and then subjected to liquid−liquid extraction, dilution and liquid chromatography with tandem mass spectrometry (LC−MS/MS) analysis using an Agilent 1290/SCIEX 5500 QTRAP system equipped with an Agilent SB-C18 reversed-phase column (Metabolon, Inc.). LC−MS/MS profiling of bile acids was conducted according to Good Clinical Practice, and assay validation included assessment for selectivity, specificity, limit of quantification accuracy and precision and stability. Fatty acid profiling was conducted according to Research Use Only specifications.

Untargeted stool metabolomics for extended bile acid analysis was carried out by Metabolon, Inc. according to Research Use Only specifications. For each sample, recovery standards were added prior to the first step in the extraction process for quality control purposes. To remove protein, to dissociate small molecules bound to protein or trapped in the precipitated protein matrix and to recover chemically diverse metabolites, proteins were precipitated with methanol under vigorous shaking for 2 minutes (Glen Mills, GenoGrinder 2000) followed by centrifugation. The resulting extract was divided into five fractions: two for analysis by two separate reversed-phase/ultra-performance liquid chromatography with tandem mass spectrometry (RP/UPLC−MS/MS) methods with positive ion mode electrospray ionization (ESI), one for analysis by RP/UPLC−MS/MS with negative ion mode ESI and one for analysis by hydrophilic interaction (HILIC)/UPLC−MS/MS with negative ion mode ESI.

We directly measured the inhibitory effect of short-chain and medium-chain fatty acids on *C. difficile* in an in vitro batch culture assay measuring *C. difficile* growth across a range of concentrations of fatty acids and three pH values both typical of a healthy colonic environment. Short-chain and medium-chain fatty acids are found in concentrations of up to 120 mM in human colonic contents, with corresponding pH ranging from 5.6 to 7.0 depending on the colonic region^59,60^. *C. difficile* was cultured in the presence of mannitol or methionine/glycine, which are two different growth substrates that *C. difficile* uses and metabolizes by different pathways in vivo^50–52^. All experiments were carried out with three different *C. difficile* ribotypes (RT-001, *Clostridioides difficile* American Type Culture Collection (ATCC) 9689; RT-060, *Clostridioides difficile* ATCC 43593; RT-087, *Clostridioides difficile* ATCC 43255) to assess strain or ribotype-specific impacts. Half-maximal inhibitory concentration (IC_50_) values were calculated from curves based on growth, quantified by measuring optical density at 600 nm (OD_600_) across eight concentrations of fatty acids. Each estimate of IC_50_ is based on the average optical density value from three replicate cultures.

VOS dose material from five batches (generated from four donors, with one donor contributing material to two separate batches) was assayed in vitro for production of DCA, LCA, butyrate, hexanoate and valerate by growing each in a single complex liquid medium across a 10-fold dilution series with eight dilutions in triplicate. Cultures were grown for 5 days, and then supernatant was collected for metabolite concentration quantification by LC−MS/MS as described above (Metabolon, Inc.). Metabolite concentrations at time 0, obtained from supernatants collected at the beginning of the experiment right after inoculation, were subtracted from final concentrations to account for the composition of the media. Dilutions with the highest concentration for each metabolite are reported.

### Statistical analyses

The phase 3 preplanned microbiome-related analyses included evaluation of VOS dose species engraftment, changes in species composition and bile acid concentrations in VOS as compared to placebo groups from baseline to weeks 1, 2, 8 and 24 (ref. ^12^). Preplanned analyses of microbiome compositional and targeted metabolomic for primary and secondary bile acids were performed on the safety population, and the distribution of patient engraftment and secondary bile acid measurements within treatment group were assessed with non-parametric median and interquartile range statistics. The evaluation of engraftment, changes in species composition and bile acid concentrations presented herein are post hoc analyses consistent with the preplanned statistical analysis with the following deviations. (1) The population for the preplanned microbiome analyses comprised all patients who were randomized, received any amount of study drug and who provided at least one evaluable stool sample before treatment and at least one evaluable stool sample after treatment. In these post hoc analyses, samples collected after treatment of an on-study CDI recurrence were excluded, as treatment of *C. difficile* recurrence requires antibiotics, which impact microbiome signals (Extended Data Fig. 1). These post-recurrence, post-antibiotic samples were more common in patients receiving placebo, where the recurrence rate was significantly higher. Removal of these samples enables examination of the impact of treatment without the confounding impact of antibiotics. (2) For analyses not requiring both pretreatment and posttreatment samples to derive values, both single and paired pretreatment/posttreatment stool samples were used. (3) Bile acid concentrations were compared directly as absolute concentrations instead of evaluated as change from baseline to enable clear communication of results. Comparisons to VOS phase 1, VOS phase 2 and the healthy comparator cohort, as well as fatty acid concentration measurements, were not preplanned.

Figures and statistical analyses were carried out using R version 3.6.3. *P* values reported for comparisons between VOS and placebo arms at single timepoints were generated using two-sided MWU in R using the rstatix package version 0.7.2. This included comparisons of engraftment, engraftment within prespecified subgroups (that is, age <65 years versus ≥65 years) and prior antibiotic regimen (vancomycin versus fidaxomicin), phylum-level relative abundances and concentrations of bile acids and fatty acids. Box plots used to visualize metrics show the median (central horizontal line) and interquartile range (shaded box) in each group at each timepoint; vertical bars indicate the most extreme non-outlier values (within 1.5 times the interquartile range); and crosses (+) indicate outlier values (outside 1.5 times the interquartile range).

NMDS plots were used to visualize the community composition of patient microbiome samples in the context of healthy individuals. Differences in overall community composition between treatment arms at each timepoint were evaluated with PERMANOVA tests, and sample dispersion was assessed using the PERDISP2 procedure. MetaMDS, adonis and betadispers functions in vegan version 2.5-6, respectively, were used. Differences in genus prevalence across arms were assessed using Fisherʼs exact tests adjusted for multiple hypothesis testing by controlling the false discovery rate (FDR) with the Benjamini−Hochberg procedure. Plots were generated using the following packages: dplyr version 1.1.2, ggplot2 version 3.7, ggpubr version 0.6.0 and reshape2 version 1.1.4.

### Reporting summary

Further information on research design is available in the Nature Portfolio Reporting Summary linked to this article.

## Online content

Any methods, additional references, Nature Portfolio reporting summaries, source data, extended data, supplementary information, acknowledgements, peer review information; details of author contributions and competing interests; and statements of data and code availability are available at 10.1038/s41591-025-04076-w.

## Supplementary information

Supplementary Information Supplementary Figs. 1−7 and Supplementary Tables 1−3.

Reporting Summary

## Figures and Tables

**Extended Data Table 1:**
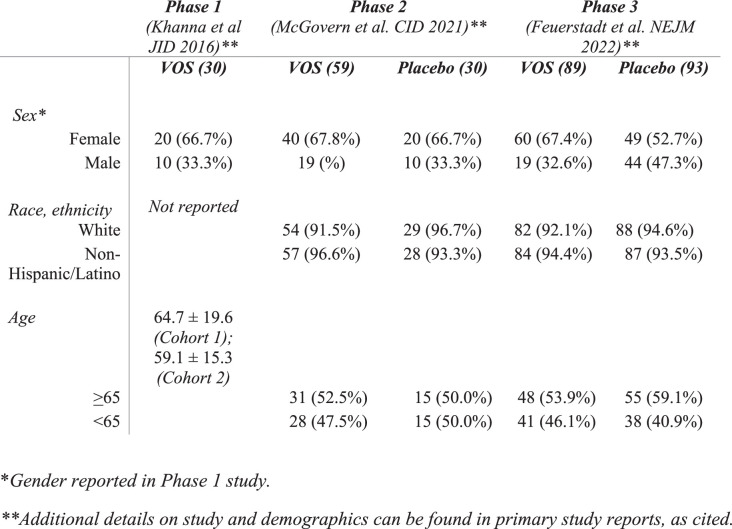
Summary demographics across studies

**Extended Data Table 2:**
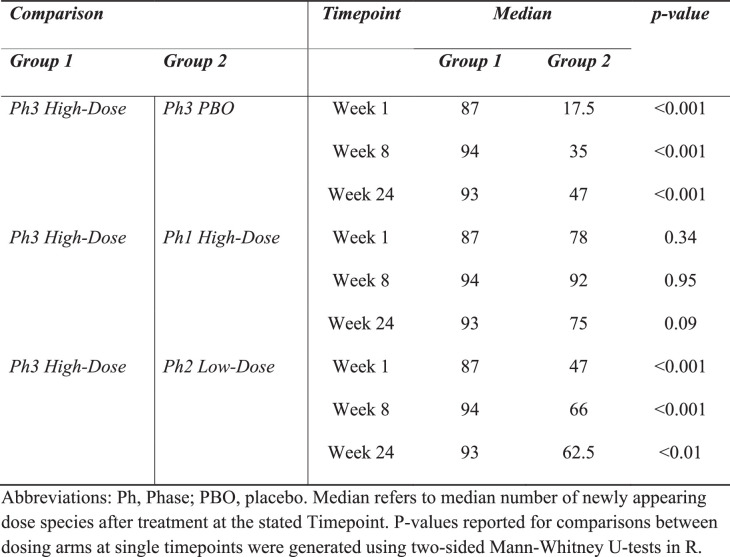
Comparison of dose species engraftment among VOS phase 1b, 2 and 3 clinical trial dosing regimens and timepoints

## Data Availability

Metabolomics, sequencing and individual-level patient data may be requested for non-commercial purposes by contacting NHScdatarequests@us.nestle.com. All requests will be reviewed by a member of the Nestlé Health Science legal team to ensure alignment with applicable patient consent agreements and regulatory requirements. Request evaluations will be completed within 4 weeks of submission. Length of time for data access will be contingent upon the requester’s research needs. The protocol and statistical analysis plan have been published (see Feuerstadt et al.^12^ and Cohen et al.^19^).
