# Supplementary material for: The impact of an oral purified microbiome therapeutic on the gastrointestinal microbiome
Source: Nat Med. 2026 Jan 5;32(1):186–96. doi: 10.1038/s41591-025-04076-w (PMC12823442; doi:10.1038/s41591-025-04076-w)
Supplement: Supplementary file 1 — Supplementary Figs. 1−7 and Supplementary Tables 1−3. [file 41591_2025_4076_MOESM1_ESM.pdf]

nature medicine

Supplementary information https://doi.org/10.1038/s41591-025-04076-w

The impact of an oral purified microbiome therapeutic on the gastrointestinal microbiome

In the format provided by the authors and unedited

Supplementary Information for: The impact of an oral purified microbiome therapeutic on the gastrointestinal microbiome

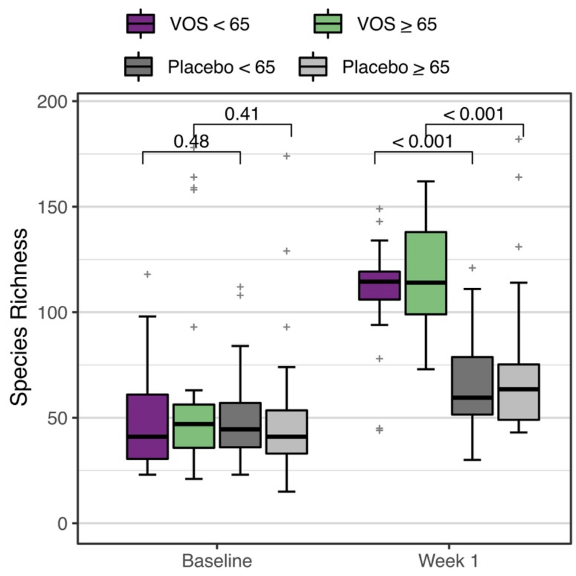

Supplementary Fig. 1: Increased species richness one week after VOS treatment irrespective of age in the phase 3 trial. Box plots indicating species richness (total number of species) observed in subject samples who were less than 65 years of age or 65 years of age or older at baseline and week one timepoints. All box plots show the median (central horizontal line) and interquartile range (shaded box) in each group at each time point; vertical bars indicate the most extreme non-outlier values (within 1.5 times the interquartile range), and crosses (+) indicate outlier values (outside 1.5 times the interquartile range). P-values indicate single comparisons of VOS vs placebo within each subpopulation and timepoint with two-sided Mann-Whitney U-tests. Sample numbers are identical to those in **Extended Data Fig. 3**.

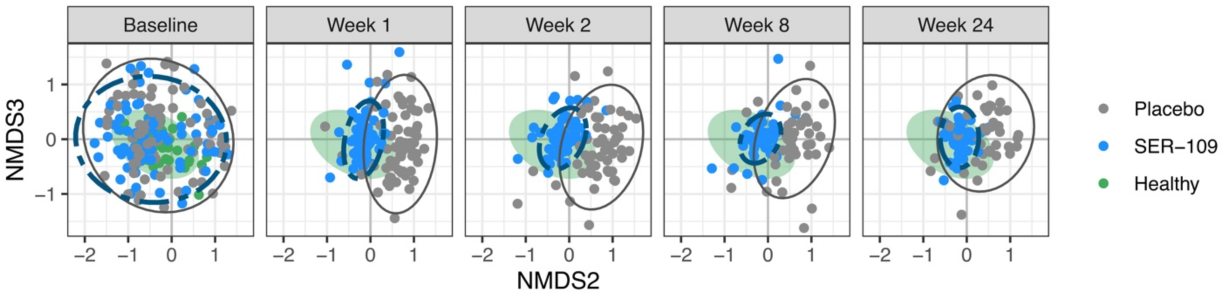

Supplementary Fig. 2: Compositional differences between VOS and placebo treated samples. Displays axes 2 and 3 of a non-metric multidimensional scaling (NMDS) plot showing Bray-Curtis dissimilarity of microbial composition between subject samples from baseline (pre-treatment) through 24 weeks post-treatment in the Phase 3 study (k = 3, stress = 0.13), including ellipses outlining 95% confidence intervals of the bivariate normal fit to each group. A cohort of 68 healthy individuals are also plotted as a reference at the pre-treatment baseline (green points), and the associated ellipse (green filled) for this healthy population was projected across all timepoints for reference. **Fig. 3** in the main text displays NMDS axes 1 and 2.

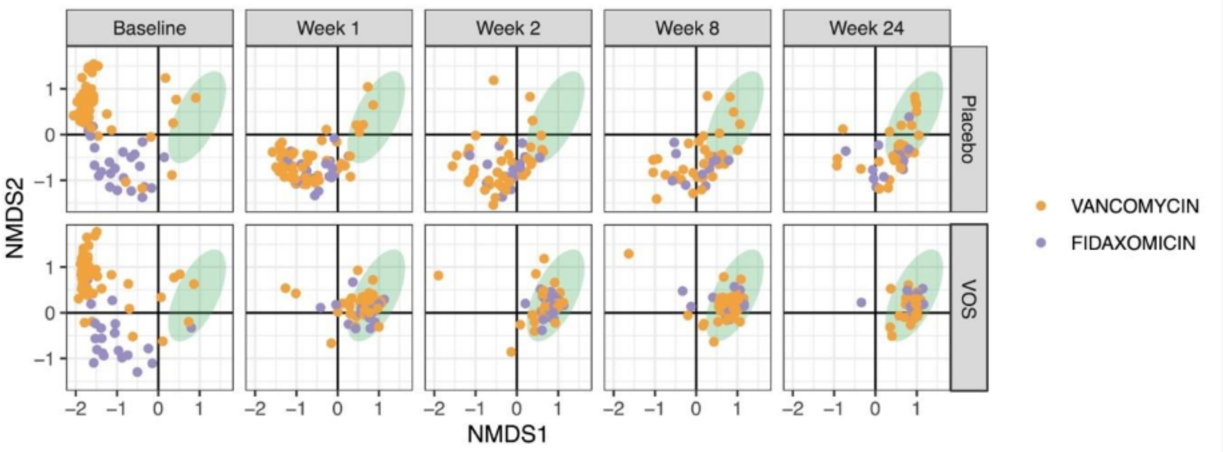

Supplementary Fig. 3: Compositional differences between standard of care antibiotic evident at baseline in the Phase 3 trial. An alternative rendering of axes 1 and 2 of the non-metric multidimensional scaling plot displayed in **Fig. 3** of the main text with samples faceted by treatment arm and re-colored to display the antibiotic used to treat each subject's qualifying episode of rCDI, from baseline (pre-treatment) through 24 weeks post-treatment in the Phase 3 study. Data ellipses (green filled) outlining the estimation of the distribution of the 68 individuals in the healthy reference group are projected onto each timepoint. Number of subjects with a sample available for at least one timepoint, for each group are as follows: VOS and Vancomycin: 60, VOS and Fidaxomicin: 23, Placebo and Vancomycin: 65, Placebo and Fidaxomicin: 24.

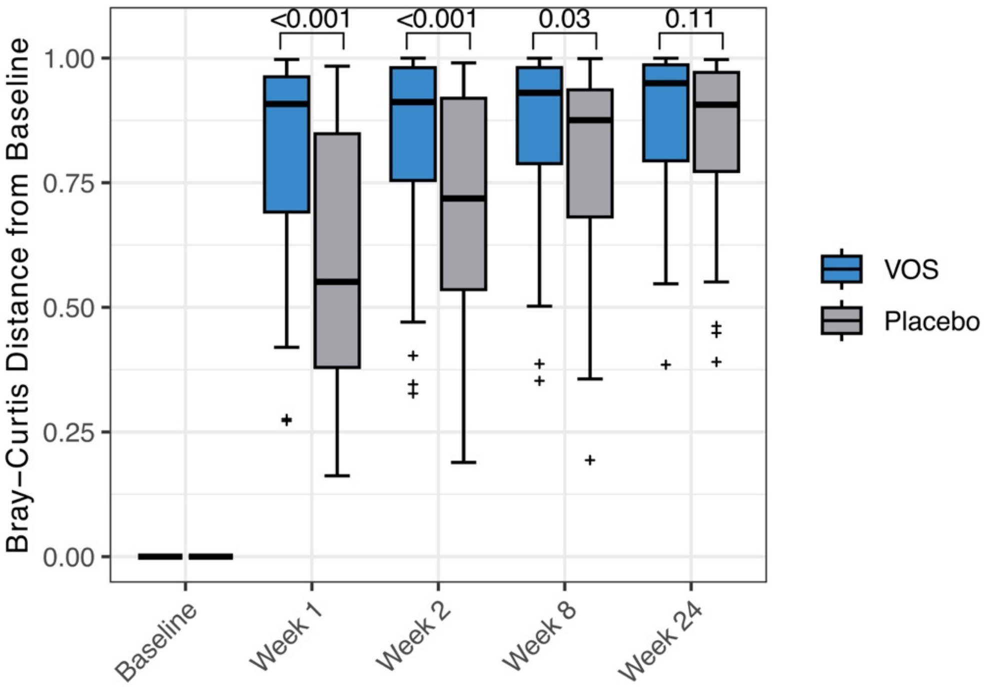

Supplementary Fig. 4. Bray-Curtis distances of each subject sample to their paired baseline sample in the Phase 3 trial. Zero indicates the two subpopulations have the same composition (they share all the species at the same relative abundances), and 1 indicates the subpopulations do not share any species. All box plots show the median (central horizontal line) and interquartile range (shaded box) in each group at each time point; vertical bars indicate the most extreme non-outlier values (within 1.5 times the interquartile range), and crosses (+) indicate outlier values (outside 1.5 times the interquartile range). P-values indicate single comparisons of distance to baseline in VOS vs placebo subjects across each timepoint with two-sided Mann-Whitney U-tests. Sample numbers as listed for the Phase 3 trial in **Fig. 1**.

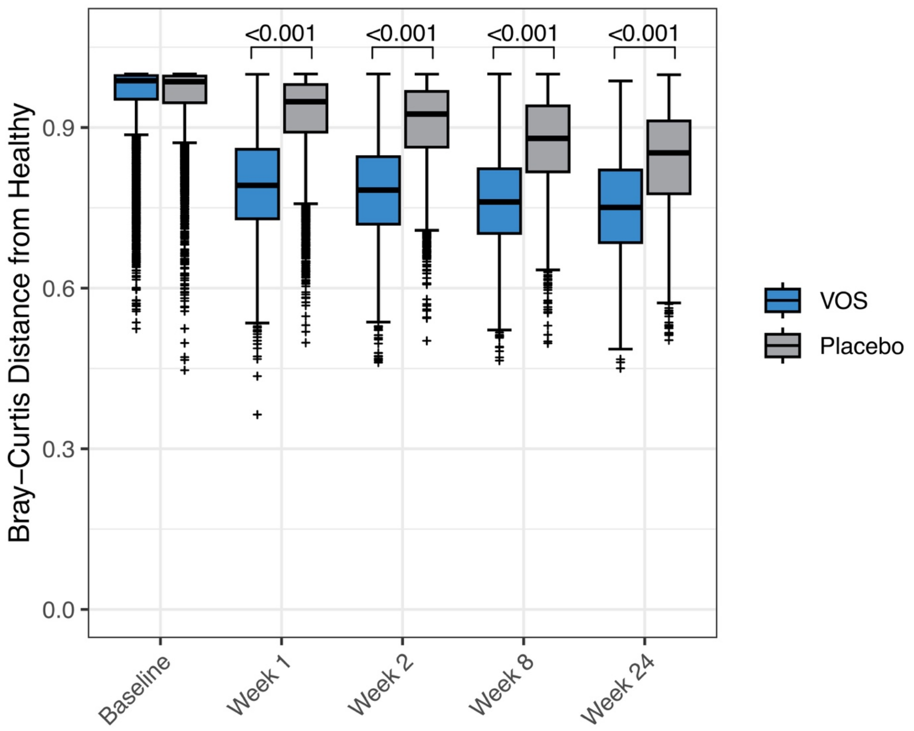

Supplementary Fig. 5: Bray-Curtis distances of all combinations of pairwise distances between subject samples to healthy cohort samples plotted in the Phase 3 trial. All box plots show the median (central horizontal line) and interquartile range (shaded box) in each group at each time point; vertical bars indicate the most extreme non-outlier values (within 1.5 times the interquartile range), and crosses (+) indicate outlier values (outside 1.5 times the interquartile range). P-values indicate single comparisons of distance to healthy subjects in VOS vs placebo subjects across each timepoint with two-sided Mann-Whitney U-tests. Data points making up the boxplot are all combinations between samples listed at each time point for the Phase 3 trial in **Fig. 1** and the 68 healthy cohort samples.

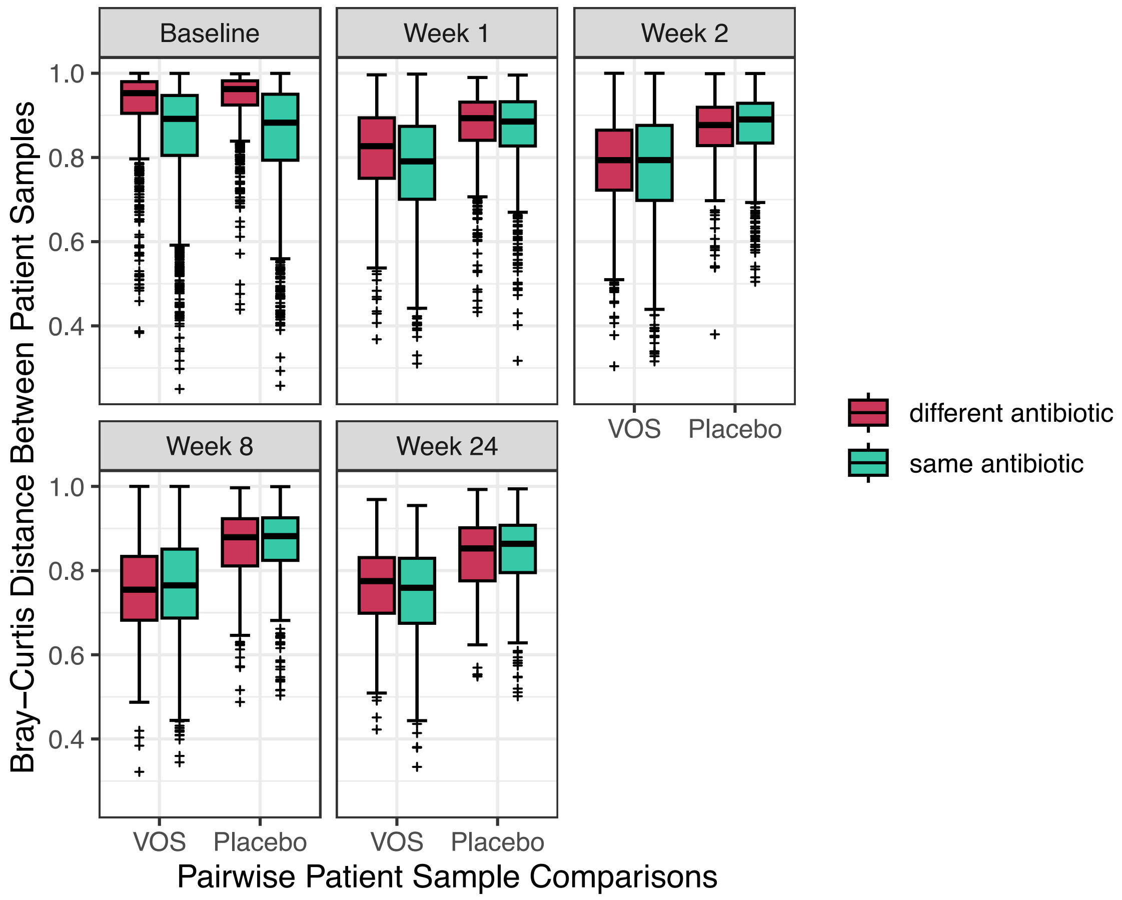

Supplementary Fig. 6: Bray-Curtis distances of all pairs of subject samples within a time point and arm in the Phase 3 trial. Boxplots compare whether two subjects received the same or differing antibiotics before dosing. The largest distances between subjects were observed at baseline (before dosing) when comparing subjects that received different antibiotics to treat their qualifying CDI episode. All box plots show the median (central horizontal line) and interquartile range (shaded box) in each group at each time point; vertical bars indicate the most extreme non-outlier values (within 1.5 times the interquartile range), and crosses (+) indicate outlier values (outside 1.5 times the interquartile range). Data points making up the boxplot are all combinations between samples listed in **Fig. 2E**, within each timepoint.

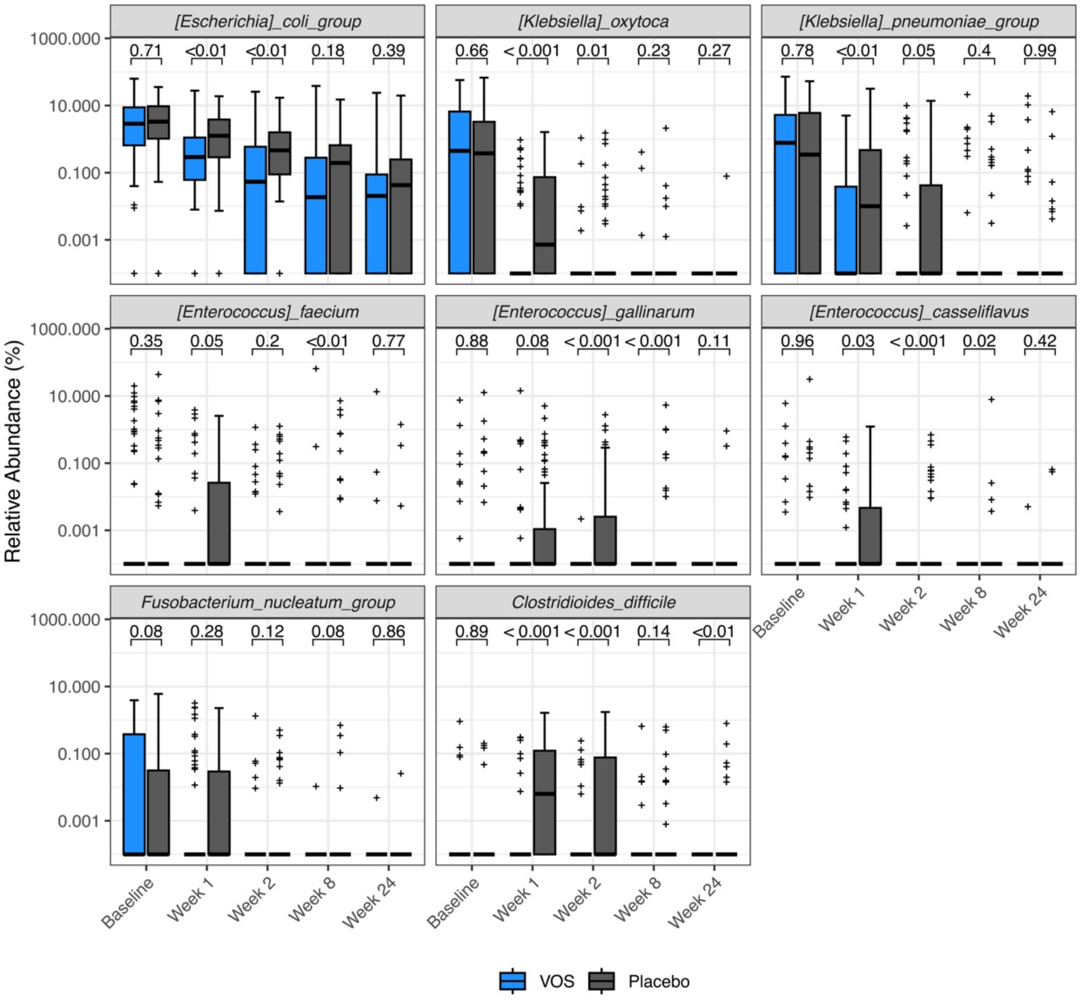

Supplementary Figure 7: Relative abundances of select bacterial species across study timepoints. Box plots indicating the percentage of selected species in each stool sample. Note, y-axis is plotted on a log scale to better view the distribution of sample abundances below 1%. All box plots show the median (central horizontal line) and interquartile range (shaded box) in each group at each time point; vertical bars indicate the most extreme non-outlier values (within 1.5 times the interquartile range), and crosses (+) indicate outlier values (outside 1.5 times the interquartile range). P-values indicate single comparisons with two-sided Mann-Whitney U-tests.

Supplementary Table 1. Phase 1 and Phase 2 trial sample inclusion by treatment arm and timepoint.

			Baseline	Week 1	Week 8	Week 24
Phase 1b (SERES-001)	VOS	Sample not provided	2	6	10	10
	VOS	Collected after recurrence	0	2	4	3
	VOS	Low Sequencing depth	1	0	0	0
	VOS	Used in Analysis	27	22	20	20
Phase 2b (SERES-004)	VOS	Sample not provided	12	15	24	37
	VOS	Collected after recurrence	0	6	9	9
	VOS	Low Sequencing depth	3	3	3	3
	VOS	Used in Analysis	44	35	23	10
	Placebo	Sample not provided	4	4	10	16
	Placebo	Collected after recurrence	0	3	5	5
	Placebo	Low Sequencing depth	0	0	0	1
	Placebo	Used in Analysis	26	23	15	8

Supplementary Table 2: Log10 fold change in primary and secondary bile acids using global metabolomics. Positive log10(fold change) values indicate higher concentrations in patients that received VOS and negative values indicate higher concentrations in patients that received placebo. P-value calculated with Mann-Whitney U-tests. False discovery rate calculated using the Benjamini-Hochberg procedure.

Classification	Chemical Name	Timepoint	log10(fold change)	p-value	False Discovery Rate
Primary	cholate	Baseline	-0.19	0.08	0.43
Primary	cholate	Week 1	-1.1	< 0.001	< 0.001
Primary	cholate	Week 2	-0.95	< 0.001	< 0.001
Primary	cholate	Week 8	-0.43	0.05	0.13
Primary	cholate	Week 24	-0.51	0.04	0.22
Primary	glycocholate	Baseline	-0.05	0.6	0.76
Primary	glycocholate	Week 1	-0.53	< 0.001	< 0.001
Primary	glycocholate	Week 2	-0.44	< 0.001	0.002
Primary	glycocholate	Week 8	-0.29	0.01	0.04
Primary	glycocholate	Week 24	-0.37	0.03	0.22
Primary	chenodeoxycholate	Baseline	-0.1	0.08	0.43
Primary	chenodeoxycholate	Week 1	-1.04	< 0.001	0.001
Primary	chenodeoxycholate	Week 2	-0.61	0.02	0.04
Primary	chenodeoxycholate	Week 8	-0.49	0.09	0.19
Primary	chenodeoxycholate	Week 24	-0.44	0.09	0.28
Primary	glycochenodeoxycholate	Baseline	-0.22	0.25	0.49
Primary	glycochenodeoxycholate	Week 1	-0.39	< 0.001	< 0.001
Primary	glycochenodeoxycholate	Week 2	-0.33	0.006	0.01
Primary	glycochenodeoxycholate	Week 8	-0.24	0.03	0.08
Primary	glycochenodeoxycholate	Week 24	-0.35	0.06	0.23
Primary	taurochenodeoxycholate	Baseline	0.25	0.38	0.56
Primary	taurochenodeoxycholate	Week 1	-0.1	0.66	0.73
Primary	taurochenodeoxycholate	Week 2	0.08	0.83	0.88
Primary	taurochenodeoxycholate	Week 8	-0.17	0.4	0.51
Primary	taurochenodeoxycholate	Week 24	0.11	0.79	0.87
Primary	taurocholate	Baseline	0.28	0.21	0.49
Primary	taurocholate	Week 1	-0.13	0.48	0.56
Primary	taurocholate	Week 2	0.12	0.76	0.82
Primary	taurocholate	Week 8	0.07	0.5	0.61
Primary	taurocholate	Week 24	0.09	0.85	0.9

Primary	cholate sulfate	Baseline	-0.3	0.1	0.43
Primary	cholate sulfate	Week 1	-0.6	< 0.001	0.001
Primary	cholate sulfate	Week 2	-0.58	0.009	0.02
Primary	cholate sulfate	Week 8	-0.75	0.001	0.01
Primary	cholate sulfate	Week 24	-0.27	0.22	0.41
Primary	glycocholate sulfate	Baseline	0.06	0.88	0.93
Primary	glycocholate sulfate	Week 1	-0.05	0.35	0.44
Primary	glycocholate sulfate	Week 2	-0.1	0.24	0.34
Primary	glycocholate sulfate	Week 8	-0.06	0.32	0.47
Primary	glycocholate sulfate	Week 24	-0.02	0.8	0.87
Primary	glycochenodeoxycholate 3-sulfate	Baseline	-0.41	0.11	0.43
Primary	glycochenodeoxycholate 3-sulfate	Week 1	-0.58	< 0.001	0.001
Primary	glycochenodeoxycholate 3-sulfate	Week 2	-0.69	< 0.001	< 0.001
Primary	glycochenodeoxycholate 3-sulfate	Week 8	-0.28	0.12	0.23
Primary	glycochenodeoxycholate 3-sulfate	Week 24	-0.23	0.18	0.39
Primary	glycocholate glucuronide (1)	Baseline	-0.02	0.84	0.91
Primary	glycocholate glucuronide (1)	Week 1	0.02	0.91	0.91
Primary	glycocholate glucuronide (1)	Week 2	0	0.89	0.92
Primary	glycocholate glucuronide (1)	Week 8	0.05	0.38	0.49
Primary	glycocholate glucuronide (1)	Week 24	0.01	0.88	0.91
Primary	glycochenodeoxycholate glucuronide (1)	Baseline	-0.15	0.53	0.71
Primary	glycochenodeoxycholate glucuronide (1)	Week 1	0.03	0.78	0.82
Primary	glycochenodeoxycholate glucuronide (1)	Week 2	-0.02	0.98	0.98
Primary	glycochenodeoxycholate glucuronide (1)	Week 8	0.06	0.99	0.99
Primary	glycochenodeoxycholate glucuronide (1)	Week 24	-0.2	0.06	0.23
Primary	glyco-beta-muricholate**	Baseline	-0.01	0.91	0.94
Primary	glyco-beta-muricholate**	Week 1	0.12	0.32	0.42
Primary	glyco-beta-muricholate**	Week 2	0.04	0.68	0.77
Primary	glyco-beta-muricholate**	Week 8	0.11	0.32	0.47
Primary	glyco-beta-muricholate**	Week 24	-0.06	0.44	0.63
Primary	cholic acid glucuronide	Baseline	-0.11	0.31	0.51
Primary	cholic acid glucuronide	Week 1	0.02	0.86	0.87
Primary	cholic acid glucuronide	Week 2	-0.01	0.98	0.98
Primary	cholic acid glucuronide	Week 8	0.03	0.56	0.67
Primary	cholic acid glucuronide	Week 24	-0.08	0.2	0.41
Primary	chenodeoxycholic acid sulfate (1)	Baseline	-0.18	0.27	0.49
Primary	chenodeoxycholic acid sulfate (1)	Week 1	-0.15	0.45	0.53
Primary	chenodeoxycholic acid sulfate (1)	Week 2	-0.43	0.05	0.08
Primary	chenodeoxycholic acid sulfate (1)	Week 8	-0.12	0.64	0.71

Primary	chenodeoxycholic acid sulfate (1)	Week 24	-0.3	0.21	0.41
Primary	chenodeoxycholic acid sulfate (2)	Baseline	-0.2	0.16	0.44
Primary	chenodeoxycholic acid sulfate (2)	Week 1	-0.78	0.008	0.01
Primary	chenodeoxycholic acid sulfate (2)	Week 2	-0.56	0.04	0.07
Primary	chenodeoxycholic acid sulfate (2)	Week 8	-0.9	0.01	0.04
Primary	chenodeoxycholic acid sulfate (2)	Week 24	-0.33	0.26	0.46
Secondary	deoxycholate	Baseline	0.12	0.32	0.51
Secondary	deoxycholate	Week 1	0.92	< 0.001	< 0.001
Secondary	deoxycholate	Week 2	0.8	< 0.001	0.002
Secondary	deoxycholate	Week 8	0.45	0.02	0.07
Secondary	deoxycholate	Week 24	0.14	0.48	0.66
Secondary	lithocholate	Baseline	-0.09	0.1	0.43
Secondary	lithocholate	Week 1	1.34	< 0.001	< 0.001
Secondary	lithocholate	Week 2	1.16	< 0.001	0.001
Secondary	lithocholate	Week 8	0.48	0.02	0.07
Secondary	lithocholate	Week 24	0.26	0.04	0.22
Secondary	ursodeoxycholate	Baseline	-0.26	0.12	0.43
Secondary	ursodeoxycholate	Week 1	-0.48	< 0.001	< 0.001
Secondary	ursodeoxycholate	Week 2	-0.45	< 0.001	0.002
Secondary	ursodeoxycholate	Week 8	-0.31	0.03	0.07
Secondary	ursodeoxycholate	Week 24	-0.15	0.62	0.75
Secondary	dehydrocholic acid	Baseline	-0.04	0.77	0.86
Secondary	dehydrocholic acid	Week 1	-0.44	< 0.001	< 0.001
Secondary	dehydrocholic acid	Week 2	-0.38	< 0.001	< 0.001
Secondary	dehydrocholic acid	Week 8	-0.4	< 0.001	0.003
Secondary	dehydrocholic acid	Week 24	-0.08	0.02	0.22
Secondary	taurodeoxycholate	Baseline	0.04	0.71	0.85
Secondary	taurodeoxycholate	Week 1	0.49	0.004	0.007
Secondary	taurodeoxycholate	Week 2	0.51	0.009	0.02
Secondary	taurodeoxycholate	Week 8	0.54	0.005	0.02
Secondary	taurodeoxycholate	Week 24	0.3	0.14	0.32
Secondary	glycodeoxycholate	Baseline	0.03	0.28	0.49
Secondary	glycodeoxycholate	Week 1	0.46	< 0.001	< 0.001
Secondary	glycodeoxycholate	Week 2	0.64	< 0.001	< 0.001
Secondary	glycodeoxycholate	Week 8	0.29	0.1	0.21
Secondary	glycodeoxycholate	Week 24	-0.02	0.79	0.87
Secondary	3-dehydrocholate	Baseline	-0.33	0.06	0.43
Secondary	3-dehydrocholate	Week 1	-1.48	< 0.001	< 0.001
Secondary	3-dehydrocholate	Week 2	-1.21	< 0.001	< 0.001

Secondary	3-dehydrocholate	Week 8	-1	< 0.001	0.004
Secondary	3-dehydrocholate	Week 24	-0.48	0.03	0.22
Secondary	7-ketodeoxycholate	Baseline	-0.19	0.27	0.49
Secondary	7-ketodeoxycholate	Week 1	-1.36	< 0.001	< 0.001
Secondary	7-ketodeoxycholate	Week 2	-1.06	< 0.001	< 0.001
Secondary	7-ketodeoxycholate	Week 8	-0.72	0.003	0.02
Secondary	7-ketodeoxycholate	Week 24	-0.46	0.05	0.23
Secondary	glycolithocholate	Baseline	-0.07	0.34	0.51
Secondary	glycolithocholate	Week 1	0.08	0.3	0.41
Secondary	glycolithocholate	Week 2	-0.05	0.63	0.75
Secondary	glycolithocholate	Week 8	-0.13	0.41	0.51
Secondary	glycolithocholate	Week 24	0.08	0.31	0.52
Secondary	taurolithocholate	Baseline	0.05	0.15	0.43
Secondary	taurolithocholate	Week 1	0.44	< 0.001	0.001
Secondary	taurolithocholate	Week 2	0.32	0.02	0.03
Secondary	taurolithocholate	Week 8	0.13	0.24	0.41
Secondary	taurolithocholate	Week 24	0.26	0.11	0.28
Secondary	dehydrolithocholate	Baseline	0.09	0.85	0.91
Secondary	dehydrolithocholate	Week 1	1.28	< 0.001	< 0.001
Secondary	dehydrolithocholate	Week 2	0.9	0.001	0.004
Secondary	dehydrolithocholate	Week 8	0.34	0.57	0.68
Secondary	dehydrolithocholate	Week 24	0.17	0.44	0.63
Secondary	6-oxolithocholate	Baseline	0.02	0.96	0.97
Secondary	6-oxolithocholate	Week 1	1.02	< 0.001	< 0.001
Secondary	6-oxolithocholate	Week 2	0.97	< 0.001	< 0.001
Secondary	6-oxolithocholate	Week 8	0.63	< 0.001	0.007
Secondary	6-oxolithocholate	Week 24	0.48	0.002	0.05
Secondary	7-ketolithocholate	Baseline	-0.15	0.28	0.49
Secondary	7-ketolithocholate	Week 1	-1.59	< 0.001	< 0.001
Secondary	7-ketolithocholate	Week 2	-1.3	< 0.001	< 0.001
Secondary	7-ketolithocholate	Week 8	-0.88	0.003	0.02
Secondary	7-ketolithocholate	Week 24	-0.59	0.05	0.23
Secondary	7,12-diketolithocholate	Baseline	-0.27	0.15	0.43
Secondary	7,12-diketolithocholate	Week 1	-0.96	< 0.001	< 0.001
Secondary	7,12-diketolithocholate	Week 2	-1.02	< 0.001	< 0.001
Secondary	7,12-diketolithocholate	Week 8	-0.96	< 0.001	< 0.001
Secondary	7,12-diketolithocholate	Week 24	-0.47	0.02	0.22
Secondary	12-dehydrocholate	Baseline	-0.12	0.68	0.85
Secondary	12-dehydrocholate	Week 1	-0.53	0.01	0.02

Secondary	12-dehydrocholate	Week 2	-0.29	0.31	0.42
Secondary	12-dehydrocholate	Week 8	-0.21	0.6	0.68
Secondary	12-dehydrocholate	Week 24	-0.18	0.57	0.72
Secondary	hyocholate	Baseline	-0.15	0.12	0.43
Secondary	hyocholate	Week 1	-0.53	< 0.001	< 0.001
Secondary	hyocholate	Week 2	-0.57	< 0.001	< 0.001
Secondary	hyocholate	Week 8	-0.36	0.005	0.02
Secondary	hyocholate	Week 24	-0.15	0.14	0.32
Secondary	glycolithocholate sulfate*	Baseline	-0.11	0.56	0.73
Secondary	glycolithocholate sulfate*	Week 1	0.1	0.34	0.43
Secondary	glycolithocholate sulfate*	Week 2	0.07	0.57	0.71
Secondary	glycolithocholate sulfate*	Week 8	0.1	0.36	0.49
Secondary	glycolithocholate sulfate*	Week 24	0.02	0.59	0.72
Secondary	taurolithocholate 3-sulfate	Baseline	0.32	0.07	0.43
Secondary	taurolithocholate 3-sulfate	Week 1	0.14	0.38	0.46
Secondary	taurolithocholate 3-sulfate	Week 2	0.21	0.08	0.13
Secondary	taurolithocholate 3-sulfate	Week 8	0.14	0.3	0.47
Secondary	taurolithocholate 3-sulfate	Week 24	0.03	0.82	0.88
Secondary	glycocholenate sulfate*	Baseline	-0.11	0.72	0.85
Secondary	glycocholenate sulfate*	Week 1	-0.71	< 0.001	< 0.001
Secondary	glycocholenate sulfate*	Week 2	-0.78	< 0.001	< 0.001
Secondary	glycocholenate sulfate*	Week 8	-0.56	0.003	0.02
Secondary	glycocholenate sulfate*	Week 24	-0.34	0.08	0.28
Secondary	taurocholenate sulfate*	Baseline	0.48	0.09	0.43
Secondary	taurocholenate sulfate*	Week 1	-0.21	0.32	0.42
Secondary	taurocholenate sulfate*	Week 2	-0.09	0.62	0.74
Secondary	taurocholenate sulfate*	Week 8	-0.32	0.11	0.22
Secondary	taurocholenate sulfate*	Week 24	-0.16	0.52	0.69
Secondary	isoursodeoxycholate	Baseline	-0.09	0.73	0.85
Secondary	isoursodeoxycholate	Week 1	-0.4	< 0.001	0.001
Secondary	isoursodeoxycholate	Week 2	-0.39	0.002	0.006
Secondary	isoursodeoxycholate	Week 8	-0.31	0.02	0.07
Secondary	isoursodeoxycholate	Week 24	-0.15	0.38	0.59
Secondary	glycoursodeoxycholate	Baseline	-0.34	0.03	0.43
Secondary	glycoursodeoxycholate	Week 1	-0.5	< 0.001	< 0.001
Secondary	glycoursodeoxycholate	Week 2	-0.49	< 0.001	0.003
Secondary	glycoursodeoxycholate	Week 8	-0.48	< 0.001	0.004
Secondary	glycoursodeoxycholate	Week 24	-0.38	0.01	0.22
Secondary	tauroursodeoxycholate	Baseline	0.18	0.3	0.51

Secondary	tauroursodeoxycholate	Week 1	-0.13	0.57	0.65
Secondary	tauroursodeoxycholate	Week 2	0.04	0.75	0.82
Secondary	tauroursodeoxycholate	Week 8	-0.07	0.83	0.87
Secondary	tauroursodeoxycholate	Week 24	-0.02	0.75	0.87
Secondary	ursocholate	Baseline	-0.24	0.08	0.43
Secondary	ursocholate	Week 1	-0.81	< 0.001	< 0.001
Secondary	ursocholate	Week 2	-0.84	< 0.001	< 0.001
Secondary	ursocholate	Week 8	-0.5	0.05	0.13
Secondary	ursocholate	Week 24	-0.32	0.11	0.28
Secondary	glycohyocholate	Baseline	-0.08	0.75	0.85
Secondary	glycohyocholate	Week 1	-0.1	0.02	0.04
Secondary	glycohyocholate	Week 2	-0.1	0.02	0.03
Secondary	glycohyocholate	Week 8	0.02	0.81	0.87
Secondary	glycohyocholate	Week 24	-0.05	0.19	0.41
Secondary	taurohyocholate*	Baseline	0.28	0.23	0.49
Secondary	taurohyocholate*	Week 1	0.07	0.68	0.74
Secondary	taurohyocholate*	Week 2	0.04	0.65	0.76
Secondary	taurohyocholate*	Week 8	-0.05	0.59	0.68
Secondary	taurohyocholate*	Week 24	0.17	0.1	0.28
Secondary	3b-hydroxy-5-cholenoic acid	Baseline	0.06	0.43	0.61
Secondary	3b-hydroxy-5-cholenoic acid	Week 1	-0.13	0.09	0.12
Secondary	3b-hydroxy-5-cholenoic acid	Week 2	-0.04	0.73	0.81
Secondary	3b-hydroxy-5-cholenoic acid	Week 8	-0.06	0.87	0.89
Secondary	3b-hydroxy-5-cholenoic acid	Week 24	0.03	0.92	0.92
Secondary	3-dehydrochenodeoxycholate	Baseline	-0.33	0.03	0.43
Secondary	3-dehydrochenodeoxycholate	Week 1	-1.25	< 0.001	< 0.001
Secondary	3-dehydrochenodeoxycholate	Week 2	-0.62	0.004	0.009
Secondary	3-dehydrochenodeoxycholate	Week 8	-0.5	0.03	0.08
Secondary	3-dehydrochenodeoxycholate	Week 24	-0.39	0.04	0.22
Secondary	deoxycholic acid 3-sulfate	Baseline	0.22	0.11	0.43
Secondary	deoxycholic acid 3-sulfate	Week 1	0.78	0.007	0.01
Secondary	deoxycholic acid 3-sulfate	Week 2	0.63	0.04	0.08
Secondary	deoxycholic acid 3-sulfate	Week 8	0.17	0.35	0.48
Secondary	deoxycholic acid 3-sulfate	Week 24	-0.3	0.39	0.59
Secondary	glycodeoxycholate 3-sulfate	Baseline	0.2	0.03	0.43
Secondary	glycodeoxycholate 3-sulfate	Week 1	0.01	0.8	0.82
Secondary	glycodeoxycholate 3-sulfate	Week 2	-0.05	0.3	0.4
Secondary	glycodeoxycholate 3-sulfate	Week 8	-0.19	0.13	0.24
Secondary	glycodeoxycholate 3-sulfate	Week 24	-0.08	0.53	0.69

Secondary	taurodeoxycholic acid 3-sulfate	Baseline	0.18	0.39	0.56
Secondary	taurodeoxycholic acid 3-sulfate	Week 1	0.15	0.03	0.05
Secondary	taurodeoxycholic acid 3-sulfate	Week 2	0.22	0.12	0.18
Secondary	taurodeoxycholic acid 3-sulfate	Week 8	0	0.75	0.82
Secondary	taurodeoxycholic acid 3-sulfate	Week 24	-0.06	0.56	0.72
Secondary	ursodeoxycholate sulfate (1)	Baseline	-0.38	0.15	0.43
Secondary	ursodeoxycholate sulfate (1)	Week 1	-0.78	0.003	0.006
Secondary	ursodeoxycholate sulfate (1)	Week 2	-0.68	0.07	0.12
Secondary	ursodeoxycholate sulfate (1)	Week 8	-0.88	0.01	0.04
Secondary	ursodeoxycholate sulfate (1)	Week 24	-0.49	0.14	0.32
Secondary	isohyodeoxycholate	Baseline	-0.11	0.22	0.49
Secondary	isohyodeoxycholate	Week 1	0.35	< 0.001	0.002
Secondary	isohyodeoxycholate	Week 2	0.3	0.004	0.01
Secondary	isohyodeoxycholate	Week 8	0.23	0.19	0.33
Secondary	isohyodeoxycholate	Week 24	0.31	0.002	0.05
Secondary	deoxycholic acid glucuronide	Baseline	-0.04	0.46	0.63
Secondary	deoxycholic acid glucuronide	Week 1	0.04	0.7	0.76
Secondary	deoxycholic acid glucuronide	Week 2	0.03	0.58	0.71
Secondary	deoxycholic acid glucuronide	Week 8	0.08	0.28	0.45
Secondary	deoxycholic acid glucuronide	Week 24	-0.02	0.92	0.92
Secondary	glycoursodeoxycholic acid sulfate (1)	Baseline	-0.3	0.04	0.43
Secondary	glycoursodeoxycholic acid sulfate (1)	Week 1	-0.45	< 0.001	< 0.001
Secondary	glycoursodeoxycholic acid sulfate (1)	Week 2	-0.54	< 0.001	< 0.001
Secondary	glycoursodeoxycholic acid sulfate (1)	Week 8	-0.18	0.02	0.05
Secondary	glycoursodeoxycholic acid sulfate (1)	Week 24	-0.11	0.22	0.41
Secondary	lithocholate sulfate (1)	Baseline	-0.05	0.19	0.48
Secondary	lithocholate sulfate (1)	Week 1	0.39	0.007	0.01
Secondary	lithocholate sulfate (1)	Week 2	0.12	0.58	0.71
Secondary	lithocholate sulfate (1)	Week 8	-0.24	0.31	0.47
Secondary	lithocholate sulfate (1)	Week 24	-0.39	0.1	0.28
Secondary	lithocholic acid sulfate (2)	Baseline	-0.25	0.13	0.43
Secondary	lithocholic acid sulfate (2)	Week 1	-0.27	0.06	0.09
Secondary	lithocholic acid sulfate (2)	Week 2	-0.47	0.09	0.14
Secondary	lithocholic acid sulfate (2)	Week 8	-0.57	0.007	0.03
Secondary	lithocholic acid sulfate (2)	Week 24	-0.49	0.06	0.23
Secondary	tauroursodeoxycholic acid sulfate (1)	Baseline	0.03	0.72	0.85
Secondary	tauroursodeoxycholic acid sulfate (1)	Week 1	-0.32	0.05	0.07
Secondary	tauroursodeoxycholic acid sulfate (1)	Week 2	-0.22	0.11	0.16
Secondary	tauroursodeoxycholic acid sulfate (1)	Week 8	-0.16	0.09	0.19

Secondary	tauroursodeoxycholic acid sulfate (1)	Week 24	-0.15	0.34	0.54
Secondary	tauroursodeoxycholic acid sulfate (2)	Baseline	0.1	0.22	0.49
Secondary	tauroursodeoxycholic acid sulfate (2)	Week 1	0.48	< 0.001	0.001
Secondary	tauroursodeoxycholic acid sulfate (2)	Week 2	0.45	0.01	0.03
Secondary	tauroursodeoxycholic acid sulfate (2)	Week 8	0.21	0.3	0.47
Secondary	tauroursodeoxycholic acid sulfate (2)	Week 24	0.17	0.45	0.63
Secondary	12-ketolithocholate	Baseline	0.19	0.34	0.51
Secondary	12-ketolithocholate	Week 1	1.69	< 0.001	< 0.001
Secondary	12-ketolithocholate	Week 2	1.6	< 0.001	< 0.001
Secondary	12-ketolithocholate	Week 8	0.83	0.07	0.17
Secondary	12-ketolithocholate	Week 24	0.29	0.1	0.28
Secondary	3-dehydrodeoxycholate	Baseline	0.17	0.34	0.51
Secondary	3-dehydrodeoxycholate	Week 1	1.46	< 0.001	< 0.001
Secondary	3-dehydrodeoxycholate	Week 2	1.24	0.005	0.01
Secondary	3-dehydrodeoxycholate	Week 8	0.59	0.86	0.89
Secondary	3-dehydrodeoxycholate	Week 24	0.16	0.79	0.87
Secondary	taurochenodeoxycholic acid 3-sulfate	Baseline	0.37	0.26	0.49
Secondary	taurochenodeoxycholic acid 3-sulfate	Week 1	-0.17	0.38	0.46
Secondary	taurochenodeoxycholic acid 3-sulfate	Week 2	-0.06	0.35	0.46
Secondary	taurochenodeoxycholic acid 3-sulfate	Week 8	-0.18	0.13	0.24
Secondary	taurochenodeoxycholic acid 3-sulfate	Week 24	-0.09	0.26	0.46
Secondary	isoursodeoxycholate sulfate (1)	Baseline	-0.23	0.11	0.43
Secondary	isoursodeoxycholate sulfate (1)	Week 1	-0.26	0.03	0.04
Secondary	isoursodeoxycholate sulfate (1)	Week 2	-0.8	< 0.001	< 0.001
Secondary	isoursodeoxycholate sulfate (1)	Week 8	-0.55	0.002	0.01
Secondary	isoursodeoxycholate sulfate (1)	Week 24	-0.5	0.03	0.22
Secondary	deoxycholic acid 12-sulfate*	Baseline	0.05	0.97	0.97
Secondary	deoxycholic acid 12-sulfate*	Week 1	1.24	< 0.001	< 0.001
Secondary	deoxycholic acid 12-sulfate*	Week 2	0.58	0.07	0.11
Secondary	deoxycholic acid 12-sulfate*	Week 8	0.22	0.34	0.48
Secondary	deoxycholic acid 12-sulfate*	Week 24	-0.35	0.33	0.54

Supplementary Table 3. List of Clinical Sites and IRBs for the Phase 3 Study.

	Site PI Member Last Name	Site PI Member First Name	Site Member Organization	Site Member Address	Site City	Site Region / State
WCG IRB Copernicus Group IRB One Triangle Drive Suite 100 Durham, North Carolina 27713 United States	Hemaidan	Ammar	Advanced Medical Research Center	1690 Dunlawton Avenue	Port Orange	Florida
WCG IRB	Johnson	Kenneth	Kinston Medical Specialist, P.A.	702 Doctors drive	Kinston	NC
WCG IRB	Kim	Suzy	Gastroenterology Associates, PA	200 Patewood Drive	Greenvill e	South Carolina
Georgetown University IRB	Kumar	Princy	Georgetown University Hospital	3800 Reservoir Rd NW	Washingt on	District Of Columbia
WCG IRB	Misra	Bharat	Jacksonville Center for Endoscopy - Southside ; Borland Groover Clinic	4800 Belfort Road	Jacksonvi lle	Florida
WCG IRB	Nathan	Richard	Snake River Research, Pllc	2900 Cortez Avenue	Idaho Falls	Idaho
WCG IRB	Nathan	Richard	Snake River Research, Pllc	2900 Cortez Avenue	Idaho Falls	Idaho
WCG IRB	Nathan	Richard	Snake River Research, Pllc	2900 Cortez Avenue	Idaho Falls	Idaho
Department of Heatlh Affairs Northern California Health care System (IRB / Human	Nguyen	Hien	East Bay Institute for Research & Education-VA Northern California Health Care System	10535 Hospital Way, VISN21	Mather	California
WCG IRB	Pardi	Darrell	Mayo Clinic	200 First Street SW	Rochester	Minnesot a

WCG IRB	Preston	Krista	Westlake Medical Research	550 Saint Charles Drive	Thousand Oaks	California
WCG IRB	Pullman	John	Mercury Street Medical Group, PLLC	300 West Mercury Street	Butte	Montana
WCG IRB	Sims	Matthew	Beaumont Hospital-Royal Oak	3811 West 13 Mile	Royal Oak	Michigan
WCG IRB	Williams	Jeffrey	Summit Clinical Research, LLC	3320 Old Jefferson Road, Bldg. 400	Athens	Georgia
WCG IRB	Wofford	Scott	Arkansas Gastroenterology, P.A.	3401 Springhill Drive	North Little Rock	Arkansas
WCG IRB	Delgado	Belkis	San Marcus Research Clinic, Inc.	5941 Northwest 173 Drive	Miami	Florida
Regional Health Command Central (RHC-C)	Gancayco	John	San Antonio Military Medical Center (SAMMC) (Brooke Army Medical Center (BAMC))	3551 Roger Brooke Dr	San Antonio	Texas
WCG IRB	Golan	Yoav	Tufts Medical Center, Inc.	800 Washington Street, Box 817	Boston	Massachusetts
WCG IRB	Hardi	Robert	MGG Group Co., Inc., Chevy Chase Clinical Research	5550 Friendship Blvd	Chevy Chase	Maryland
WCG IRB	Korman	Louis	Metropolitan Gastroenterology Group, PC- Chevy Chase Clinical Research	5550 Friendship Boulevard	Chevy Chase	Maryland
WCG IRB	Barish	Charles	Wake Research Associates, LLC	3100 Duraleigh Road	Raleigh	North Carolina
WCG IRB	Harper	Wayne	Wake Research Associates, LLC	3100 Duraleigh Road	Raleigh	North Carolina
University of Massachusetts Medical School (IRB)	Houghton	JeanMarie	University of Massachusetts Memorial Medical Center	55 Lake Avenue North	Worcester	Massachusetts
WCG IRB	Odio	Alberto	Alta California Medical Group	2925 N. Sycamore Drive	Simi Valley	California
WCG IRB	Oubre	Benton	Benton Oubre, Md	9103 Jefferson Highway, 2nd Floor	Baton Rouge	Louisiana

WCG IRB	Kraft	Colleen	Emory University Hospital	1364 Clifton Road, NE	Atlanta	Georgia
University of Iowa Hospitals (IRB)	AssoulineDayan	Yehudith	University Of Iowa Hospital & Clinics	200 Hawkins Drive	Iowa City	Iowa
VA Western New York Healthcare System (IRB)	Berenson	Charles	Va Western New York Healthcare System	3495 Bailey Avenue, Oncology 111H	Buffalo	New York
WCG (IRB)	Ephtimios	Issa	Avanza Medical Research Center	4700 Bayou	Pensacola	Florida
Partners Human Research (IRB)	Hohmann	Elizabeth	Massachusetts General Hospital	55 Fruit Street	Boston	Massachu setts
WCG IRB	Smith	Kent	Advanced Clinical ResearchGut Whisperer	1543 W. 12600 S.	Riverton	Utah
Advarra (IRB)	Tan	Michael	Summa Center for Clinical Trials	75 Arch Street	Akron	Ohio
WCG IRB	Weinstock	Leonard	Sundance Clinical Research, LLC	711 Old Ballas Road	St Louis	Missouri
WCG IRB	Hansen	Val	Dr.Val R. Hansen MD, Office of	425 Medical Drive, Suite 110	Bountiful	Utah
Henry Ford Health System (IRB)	Ramesh	Mayur	Henry Ford Health Hospital	2799 West Grand Boulevard, Pallister place T139	Detroit	Michigan
WCG IRB	Naqvi	Syed	University Of MissouriColumbia	115 Business Loop 70 West	Columbia	Missouri
WCG IRB	Liu	Edward	Jersey Shore University Medical Center	1945 State Highway 33	Neptune	New Jersey
WCG IRB	Georgetson	Michael	Guthrie Medical Group, PC	One Guthrie Square	Sayre	Pennsylva nia
WCG IRB	Sheikh	Aasim	Gastrointestinal Specialists of Georgia, PC	711 Canton Rd.	Marietta	Georgia
WCG IRB	Feuerstadt	Paul	Gastroenterology Center of Connecticut	2200 Whitney Avenue	Hamden,	Connectic ut
Cleveland Clinic IRB	Garcia-Diaz	Julia	Ochsner Clinic Foundation	1514 Jefferson Highway	New Orleans	Louisiana
WCG IRB	Pulsipher	Dan	Asclepes Research Centers	8425 Balm St.	Weeki Wachee	Florida

University of California, Davis IRB Administration	Cohen	Stuart	UC Davis Medical Center	4150 V Street	Sacramento	California
	Gumber	Subhash	Carolina's GI Research	2601 Lake Drive	Raleigh	North Carolina
WCG IRB	Stanciu	Sebastian	Jacksonville Center for Clinical Research	4085 University Boulevard South	Jacksonville	Florida
WCG IRB	Arimie	Calin	California Medical Research Associates Inc	18531 Roscoe Boulevard	Northridge	California
WCG IRB	Andrade	Gladys	Broward Research Center	1900 North University Drive	Pembroke Pines	Florida
University of Kansas Medical Center IRB	O'Marro	Steven	Springfield Clinic - Clinical Research	1130 South 6th Street	Springfield	Illinois
University of Kansas Medical Center IRB	Esfandyari	Tuba	University of Kansas Medical Center	3901 Rainbow Boulevard, Mail Stop 3007	Kansas City	Kansas
WCG IRB	Moya	Jaynier	Pines Care Research Center, LLC	501 NW 103 Avenue	Pembroke Pines	Florida
WCG IRB	Osiyemi	Olayemi	Triple O Research Institute PA	2580 Metrocentre boulevard	West Palm Beach	Florida
WCG IRB	Ritter	Timothy	Texas Digestive Disease Consultants	505 South Nolen Drive	Southlake	Texas
Christiana Care IRB	Bacon	Alfred	Christiana Care Cardiology Consultants, PA	4755 OgletownStanton Road	Newark	Delaware
WCG IRB	Baird	Ian Mcnicol	Remington Davis	1335 Dublin Road	Columbus	Ohio
WCG IRB	Colman	Ronald	Gulf Coast Research Group, LLC	4100 S. Shepard Dr.	Houston	Texas
Marshfield Clinic Research Institute IRB	Hall	Matthew	Marshfield Clinic	1000 North Oak Avenue	Marshfield	Wisconsin
Loyola University Medical Center	Hecht	Gail	Loyola University Medical Center (LUMC)	2160 South 1st Ave, Maguire Building	Maywood	Illinois
WCG IRB	Patel	Meenakshi	Valley Medical Research	6611 Clyo Road	Centerville	Ohio

WCG IRB	Mehmood	Tariq	American Research, LLC	1035 Wall Street	Jeffersonv ille	Indiana
WCG IRB	Haaksma	James	Onsite Clinical Solutions	260 Merrimon Avenue Suite 101	Asheville	North Carolina
WCG IRB	Hernandez	Liliam	LA SALUD RESEARCH CLINIC	8415 SW 24 ST SUITE 203	MIAMI	Florida
WCG IRB	Adams	Atoya	AB Clinical Trials	2110 East Flamingo Road, Suite 103	Las Vegas	Nevada
University of Virginia Institutional Review Board for Health Sciences Research IRB-HSR	Behm	Brian	University of Virginia Medical Center (UVAMC)	1215 Lee St, BOX 800170	Charlottes ville	Virginia
WCG IRB	Welton	Thomas	Cotton-O'Neal Clinical Research Center Digestive Health	720 South West Lane Street	Topeka	Kansas
WCG IRB	Welker	James	Anne Arundel Medical Center	2001 Medical Parkway	Annapolis	Maryland
WCG IRB	Beaulieu	Eva	Infinite Clinical Trials	113 Upper Riverdale Road	Riverdale	Georgia
WCG IRB	Sherman	Alex	Concorde Medical Group, PLLC	232 East 30th Street	New York	New York
WCG IRB	Okolo	Charles	Doctors Clinical Research	1151 Cleveland Avenue	East Point	Georgia
WCG IRB	Arsenescu	Razvan	Atlantic Digestive Health Institute	435 South Street	Morristo wn	New Jersey
WCG IRB	Black	Richard	Metroboston Clinical Partners, LLC	60 Dedham Ave	Needham	MA
Western Institutional Review Board 1019 39th Avenue Southeast Puyallup, WA 98374	Henry	Patricia	Carle Foundation Hospital	611 West Park Street	Urbana	Illinois
WCG IRB	Dela Llana	Alexander	MediSphere Medical Research Center, LLC	1401 Professional Boulevard	Evansvill e	Indiana
WCG IRB	Katikaneni	Shalini	Hillcrest Dallas Clinical Research Inc	9550 Forest Lane Suite 222	Dallas	Texas
WCG IRB	Kumar	Sanjeev	MD First Research	115 Whitehall Rd	Anderson	SC
WCG IRB	Mason	Raymond	Family Practice Center of Wooster, Inc.	2935 Lincoln Way	Massillon	Ohio

Bayor Scott & White Research Institutional Review Board-Gold	Vincent	Jennifer	Baylor Scott & White Health	2401 S. 31st Street	Temple	Texas
WCG IRB	Hadi	Ghassan	Empire Clinical Research	1060 E. Foothill Blvd	Upland	California
WCG IRB	Shapiro	Max	Mt. Vernon Clinical Research	744 Mt. Vernon Highway N.E.	Sandy Springs	Georgia
WCG IRB	Shapiro	Max	Metro Atlanta Gastroenterology	5669 Peachtree Dunwoody Rd	Atlanta	Georgia
WCG IRB	Kogan	Mark	Care Access Research, San Pablo	2089 Vale Rd	San Pablo	California
WCG IRB	Bangash	Ifzal	RxClinicals	4314 West Crystal Lake Road	Crystal Lake	Illinois
WCG IRB	Jazrawi	Saad	Northwest Gastroenterology Clinic, LLC	1130 Nw 22nd	Portland	Oregon
WCG IRB	Hendrix	Ernest	North Alabama Research Center, LLC	721 West Market Street	Athens	Alabama
WCG IRB	Parker	Matthew	Heritage Medicine	50 Manning Place	Birmingh am	Alabama
WCG IRB	Jamal	Mohammad Mazen	Kindred Medical Institute for Clinical Trials, LLC	854 Magnolia Ave	Corona	California
University of Miami Human Subject Research Office	Gebhard	Ralf	Univeristy of Miami / Jackson Memorial Hospital	1611 NW 12th Avenue	Miami	Florida
WCG IRB	Branin	Bruce	Empirical Research Group	4611 nw 53rd ave	Gainesvill e	Florida
Brody School Of Medicine At ECU IRB	Cook	Paul	Brody School Of Medicine At ECU	2300 Beasley Drive, 6A Doctors Park	Greenvill e	North Carolina
WCG IRB	Hanabergh	Rodolfo	American Research Medical Group	8080 West Flagler Street	Miami	Florida
WCG IRB	Husain	Syed Nasir	Revival Research Institute LLC	43303 Schoenherr Rd	Sterling Heights	Michigan
Cleveland Clinic IRB	Lashner	Bret	Cleveland Clinic - Taussig Cancer Institute	9500 Euclid Avenue, Mail Code R35	Cleveland	Ohio

WCG IRB	Lohani	Govinda	Atria Clinical Research	4020 Richards Rd	North Little Rock	Arkansas
WCG IRB	Patel	Shatishkumar	Southwest Houston Nephrology and Hypertension Clinic	8200 Wednesbury lane	Houston	Texas
WCG IRB	Eweje	Peter	East Carolina Gastroenterology	4 Office Park Drive	Jacksonville	North Carolina
WCG IRB	Heuer	Marvin	HMD Research LLC	6001 Vineland Road	Orlando	Florida
Sutter Health - Palo Alto Medical Foundation Mount IRB	Huang	Edward	Sutter Health Palo Alto Medical Foundation - Mountain View Center	701 E El Camino Real	Mountain View	California
Western IRB (WIRB)	Rodriguez	Martin	University Of Alabama At Birmingham School Of Medicine - Alabama Vaccine Research Clinic	908 20th Street South	Birmingham	Alabama
WCG IRB	Gupta	Anubha	Southeast Clinical Research, Llc.	304 Ne 1st. Street	Chiefland	Florida
WCG IRB	Bogdanovich	Tatiana	University Of Pittsburgh	200 Lothrop Street	Pittsburgh	Pennsylvania
Western IRB (WIRB)	Rao	Satish	Georgia Regents University	1120 15th Street, AD2238	Augusta	Georgia
Reading Hospital Institutional Review Board	Powell	Debra	Reading Hospital & Medical Center	301 South, 7th Avenue	West Reading	Pennsylvania
WCG IRB	Coates	Allan	Gastroenterology Associates of Western Michigan, PLC d.b.a. West Michigan Clinical Research Center	2093 Health Drive Southwest	Wyoming	Michigan
WCG IRB	Gentry	Andrew	Bozeman Health Deaconess Hospital d/b/a Bozeman Health Clinical Research	950 Stoneridge Drive	Bozeman	MT
WCG IRB	Wilson	Jason	Charlotte Gastroenterology & Hepatology, PLLC	2015 Randolph Road, Suite 208	Charlotte	North Carolina

WCG IRB	Rai	Shiwali	Urgent Care Clinical Trials at City Doc Urgent Care	2909-B McKinney Avenue	Dallas	Texas
WCG IRB	Boren	Kenneth	Phoenician Centers for Research & Innovation (PCRI)	202 E. Earll Street	Phoenix	Arizona
South Shore Hospital IRB	Ellerin	Todd	South Shore Hospital/Director of Infectious Disease (Weymouth)	55 Fogg Road	Weymout h	Massachu setts
WCG IRB	Choi	Myung	Gastroenterology Associates, PC (Elligo Clinic Infrastructure Network)	7915 Lake Manassas Drive	Gainesvill e	Virginia
WCG IRB	Dulitz	David	Clinical Trials Management, LLC	3801 Houma Blvd	Metairie	Louisiana
WCG IRB	Valle	Emil	The Gastroenterology Group, PC (Elligo Clinic Infrastructure Network)	1939 Roland Clarke Place	Restin	Virginia
University of Chicago IRB 5751 South Woodlawn Avenue 2nd Floor	Sakuraba	Atsushi	The University of Chicago Medical Center	5758 South Maryland Avenue	Chicago	Illinois
WCG IRB	De Beixedon	John	Havana Research Institute LLC.	675 S. Arroyo Pkwy	Pasadena	California
Vancouver Island Health Authority Clinical Research Ethics Board (CREB)	Lee	Christine	Royal Jubilee Hospital, Vancouver Island Health Authority (Viha)	1952 Bay Street	Victoria	British Columbia
Advarra Central REB (Central Site)	Grimard	Doria	Q & T Research Chicoutimi	412 boulevard du Saguenay est	Chicouti mi	Quebec

Institutional Research Ethics Board, Office of Human Research Ethics	Silverman	Michael	St. Joseph's Health Care London - Infectious Diseases Care Program	268 Grosvenor Street B3-030	London	Ontario
The University of British Columbia (UBC) Clinical Research Ethics Board	Steiner	Theodore	Gordon & Leslie Diamond Health Care Centre	2775 Laurel Street	Vancouve r	British Columbia
Conjoint Health Research Ethics Board (CHREB), University of Calgary	Louie	Thomas	Alberta Health Services (AHS) - Foothills Medical Centre (FMC)	1403 29 St Nw	Calgary	Alberta
