# Supplementary material for: The impact of an oral purified microbiome therapeutic on the gastrointestinal microbiome
Source: Nat Med. 2026 Jan 5;32(1):186–96. doi: 10.1038/s41591-025-04076-w (PMC12823442; doi:10.1038/s41591-025-04076-w)
Supplement: Supplementary file 2 — Reporting Summary [file 41591_2025_4076_MOESM2_ESM.pdf]

nature portfolio

Corresponding author(s): Christopher Ford

Last updated by author(s): October 22, 2025

Reporting Summary

Nature Portfolio wishes to improve the reproducibility of the work that we publish. This form provides structure for consistency and transparency in reporting. For further information on Nature Portfolio policies, see our Editorial Policies and the Editorial Policy Checklist.

Statistics

For all statistical analyses, confirm that the following items are present in the figure legend, table legend, main text, or Methods section.

n/a	Confirmed	
☐	☒	The exact sample size (*n*) for each experimental group/condition, given as a discrete number and unit of measurement
☐	☒	A statement on whether measurements were taken from distinct samples or whether the same sample was measured repeatedly
☐	☒	The statistical test(s) used AND whether they are one- or two-sided<br>*Only common tests should be described solely by name; describe more complex techniques in the Methods section.*
☐	☒	A description of all covariates tested
☐	☒	A description of any assumptions or corrections, such as tests of normality and adjustment for multiple comparisons
☐	☒	A full description of the statistical parameters including central tendency (e.g. means) or other basic estimates (e.g. regression coefficient) AND variation (e.g. standard deviation) or associated estimates of uncertainty (e.g. confidence intervals)
☐	☒	For null hypothesis testing, the test statistic (e.g. *F*, *t*, *r*) with confidence intervals, effect sizes, degrees of freedom and *P* value noted<br>*Give P values as exact values whenever suitable.*
☒	☐	For Bayesian analysis, information on the choice of priors and Markov chain Monte Carlo settings
☒	☐	For hierarchical and complex designs, identification of the appropriate level for tests and full reporting of outcomes
☐	☒	Estimates of effect sizes (e.g. Cohen's *d*, Pearson's *r*), indicating how they were calculated

Our web collection on statistics for biologists contains articles on many of the points above.

Software and code

Policy information about availability of computer code

Data collection	Electronic data capture (EDC) system used for data collection was iMedidata Rave 2016.5.2.
Data analysis	We used open source MetaPhlan2 and Strainphlan version 4 software with custom taxonomic markers.<br><br>All statistical analyses and figures were generated in R 3.6.3 with packages: dplyr version 1.1.2, ggplot2 version 3.7, ggpubr version 0.6.0, reshape2 version 1.1.4.rstatix version 0.7.2, vegan version 2.5-6

For manuscripts utilizing custom algorithms or software that are central to the research but not yet described in published literature, software must be made available to editors and reviewers. We strongly encourage code deposition in a community repository (e.g. GitHub). See the Nature Portfolio guidelines for submitting code & software for further information.

Data

Policy information about availability of data

All manuscripts must include a data availability statement. This statement should provide the following information, where applicable:

- Accession codes, unique identifiers, or web links for publicly available datasets
- A description of any restrictions on data availability
- For clinical datasets or third party data, please ensure that the statement adheres to our policy

Metabolomics, sequencing and individual-level subject data may be requested for non-commercial purposes by contacting NHScdatarequests@us.nestle.com. All

requests will be reviewed by a member of the Nestlé Health Science Legal team to ensure alignment with applicable subject consent agreements and regulatory requirements. Request evaluations will be completed within four weeks of submission. Length of time for data access will be continent on requester's research needs. The protocol and statistical analysis plan have been published (see Feuerstadt et al.12 and Cohen et al.19)

The protocol and statistical analysis plan have been previously published/made available.

Research involving human participants, their data, or biological material

Policy information about studies with human participants or human data. See also policy information about sex, gender (identity/presentation), and sexual orientation and race, ethnicity and racism.

Reporting on sex and gender

Phase 1 (Khanna et al JID 2016)
Gender, n (%)Female 20 (66.7); Male 10 (33.3) (cohort 1 and Cohort 2 had identical percentages, and are shown combined here)

Phase 2 (McGovern et al CID 2021)
Sex, no. 109, Placebo (%): 40 (67.8); 20 (66.7)

Phase 3 (Feuerstadt et al. NEJM 2022)
Sex, no. 109, PBO (%)
Female 60 (67.4) 49 (52.7)

Sex ("Gender" in the Phase 1) was not considered in the clinical trial study design of any of the three studies. Sex was determined based on self-reporting or health records. Sex-based analyses were conducted and reported for Phase 3 ECOSPOR III primary endpoint in Berenson et al. CID 2023. Treatment effect of lower CDI recurrence in VOWST versus placebo did not differ between males and females. Therefore sex-based microbiome analyses were not executed.

Reporting on race, ethnicity, or other socially relevant groupings

Phase 1 (Khanna et al JID 2016)
Investigator sponsored study; race/ethnicity not reported

Phase 2 (McGovern et al CID 2021)
White race, no. 109, Placebo (%): 54 (91.5); 29 (96.7)
Non-Hispanic/Latino ethnicity ,no. 109, Placebo (%): 57 (96.6); 28 (93.3)

Phase 3 (Feuerstadt et al. NEJM 2022)
Race, 109, PBO no. (%) †
Asian 1 (1.1) 0 (0.0)
African American 4 (4.5) 4 (4.3)
White 82 (92.1) 88 (94.6)
Other 2 (2.2) 1 (1.1)
Ethnicity, 109, PBO, no. (%) †
Hispanic or Latino 5 (5.6) 6 (6.5)
Not Hispanic or Latino 84 (94.4) 87 (93.5)

Population characteristics

Sex, Race, ethnicity reported above.
Phase 1 (Khanna et al JID 2016)
Age, years:
Mean ± SD: 64.7 ± 19.6 (Cohort 1); 59.1 ± 15.3 (Cohort 2)
Median (minimum, maximum): 71.0 (22, 88) (Cohort 1); 58.0 (39, 83)(Cohort 2)

Phase 2 (McGovern et al CID 2021)
Age class:
<65 years (109, PBO): 28 (47.5); 15 (50.0)
≥65 years (109, PBO): 31 (52.5); 15 (50.0)

Phase 3 (Feuerstadt et al. NEJM 2022)
Age
Mean, years (SD) 65.6 (16.5) 65.5 (16.7)
Distribution, 109, PBO no. (%)
<65 years 41 (46.1) 38 (40.9)
≥65 years 48 (53.9) 55 (59.1)

Population characteristics are provided in the manuscript; other characteristics have been previously reported. Feuerstadt et al. NEJM 2022

Recruitment

Description of recruitment is reported in the manuscript as well as has been previously reported. Feuerstadt et al. NEJM 2022

Ethics oversight

The Institutional Review Board for each study site reviewed and approved the study. IRB details for the Phase 3 study are provided in the Ethics section of the methods document. The formatting of that table precludes inclusion in this document.

Note that full information on the approval of the study protocol must also be provided in the manuscript.

Field-specific reporting

Please select the one below that is the best fit for your research. If you are not sure, read the appropriate sections before making your selection.

☒ Life sciences ☐ Behavioural & social sciences ☐ Ecological, evolutionary & environmental sciences

For a reference copy of the document with all sections, see nature.com/documents/nr-reporting-summary-flat.pdf

Life sciences study design

All studies must disclose on these points even when the disclosure is negative.

Sample size

Sample sizes are reported in this manuscript.

Sample sizes for the phase 3 clinical trial were determined as described in Feuerstadt et al. NEJM 2022:

"Based on the published literature and Seres clinical trial experience, the placebo recurrence rate was estimated to be 36%. Assuming a 36% recurrence rate for the control group and a 16% recurrence rate in the SER-109 group based on the Phase 1 data, the sample size for this study would provide the following power estimates based on the fixed sequence multiple testing strategy to be implemented for this study:
• to test the null hypothesis (H1) that the relative risk (RR) of CDI recurrence of SER-109 to placebo is ≥ 1.0 vs the alternative hypothesis (Ha1) that the RR < 1.0 at a one-sided significance level of 0.025, the sample size will provide 83% power.
• If H1 is found to be statistically significant, then H2: RR ≥ 0.833 vs Ha2: RR < 0.833 will be tested at a one-sided significance level of 0.025. The sample size will provide 62% power to test H2."

For the metabolite production screen run on VOWST doses, no sample size calculation was performed. Prior development work with the metabolite production assay informed the determinatio that triplicates are sufficient to capture binary outcomes (production versus non-production of metabolites). All measured metabolite concentrations were above all replicate media control samples giving us confidence in the results.

For the inhibition of C. difficile growth assay, sample size calculations were not performed. Prior assay development work with fewer conditions informed the decision. Consistency in trends across ribotypes (n of 3) and culture conditions (3 pH levels, 2 carbon sources) suggests these sample sizes are sufficient.

Data exclusions

Stool samples were excluded from analyses if subjects experienced a CDI recurrence, or if sequencing depths for a sample did not meet quality thresholds. Patients that experience a rCDI episode may receive additional antibiotic treatment which confounds the impact of dosing on the microbiome. A minimum sequencing depth is necessary to ensure microbiome communities are sufficiently and consistently characterized. Note some subjects also did not provide stool samples for some or all time points. Breakdowns of the number of missing or excluded samples are provided in the manuscript. Sequencing depth exclusions were preplanned. Sample exclusion due to on-study CDI recurrences was not preplanned.

Replication

The metabolite production screen run on VOWST doses was conducted once, with three replicates of each experimental condition. Each replicate is plotted individually on the figure.

The inhibition of C. difficile growth by short and medium chain fatty acids was evaluated in a single experiment, run in triplicate, and with three different ribotypes of C. difficile. Replicates were consistent both within and across ribotypes, and OD measures were averaged across replicates for plotting.

Randomization

Patients were randomized to treatment arm. Description reported in other publications on the same trial.

Blinding

The study was double blind clinical trial. Description reported in other publications on the same trial.

Reporting for specific materials, systems and methods

We require information from authors about some types of materials, experimental systems and methods used in many studies. Here, indicate whether each material, system or method listed is relevant to your study. If you are not sure if a list item applies to your research, read the appropriate section before selecting a response.

Materials & experimental systems

n/a	Involved in the study
☒	☐ Antibodies
☒	☐ Eukaryotic cell lines
☒	☐ Palaeontology and archaeology
☒	☐ Animals and other organisms
☐	☒ Clinical data
☒	☐ Dual use research of concern
☒	☐ Plants

Methods

n/a	Involved in the study
☒	☐ ChIP-seq
☒	☐ Flow cytometry
☒	☐ MRI-based neuroimaging

Clinical data

Policy information about clinical studies

All manuscripts should comply with the ICMJE guidelines for publication of clinical research and a completed CONSORT checklist must be included with all submissions.

Clinical trial registration: clinicaltrials.gov

Study protocol: NCT03183128

Data collection: The trial was conducted across 56 medical sites across the United States and Canada. from July 2017 through Sept 2020. The electronic data capture (EDC) system iMedidata Rave 2016.5.2 was used for data collection. Description reported in Feuerstadt et al. NEJM 2022, a publication on the same trial that focuses on trial outcome.

Outcomes: Description reported in Feuerstadt et al. NEJM 2022, a publication on the same trial that focuses on trial outcome. CDI recurrence "was defined as onset of three or more unformed bowel movements per day over 2 consecutive days, a positive C. difficile stool toxin assay (enzyme immunoassay or cell cytotoxicity neutralization assay), an assessment by the investigator that treatment was warranted, and persistence of diarrhea until antibiotic treatment was initiated." - Feuerstadt et al. NEJM 2022,

Plants

Seed stocks: NA

Novel plant genotypes: NA

Authentication: NA
